# Pit Morphology, Dissolution Kinetics, and Gas Generation Monitored in Real Time during Localized Anodic Aluminum Corrosion

**DOI:** 10.1021/jacs.5c11352

**Published:** 2025-09-10

**Authors:** Morgan Barbey-Binggeli, Vasiliki Tileli

**Affiliations:** Institute of Materials, 27218 École Polytechnique Fédérale de Lausanne, CH-1015 Lausanne, Switzerland

## Abstract

Localized corrosion in metallic materials is a stochastic phenomenon that causes irreversible structural failure. Its initiation, which occurs at the solid–liquid interface on the nanometer scale, remains difficult to predict and challenging to characterize. Herein, we describe an experimental platform that exploits advances in electrochemical liquid-phase scanning and transmission electron microscopy (LPSEM and LPTEM) to study pitting corrosion of thin-film pure aluminum in a saline environment in real time. Galvanostatic measurements at increasing current levels showed that localized corrosion of Al begins with the appearance of blisters in parallel with nanosized pits. It progresses with the coexistence of round and fractal-like pit morphologies before transitioning to the complete fractal-like dissolution of Al at high currents. Although gas bubble formation appeared to be more pronounced at higher currents, we were able to locally probe that the gas is produced at the corrosion front, which we experimentally confirmed to be molecular hydrogen. Our findings reveal the kinetic mechanism of the early stages of localized anodic corrosion in Al, which may have more general implications for proposing corrosion resistance descriptors.

## Graphical abstract

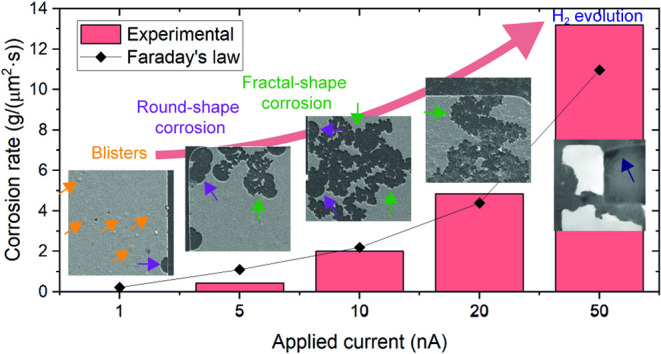

## Introduction

Metallic corrosion remains a major hindrance for structural materials^1^ underpinning the performance of a plethora of everyday applications and raising safety, environmental, and economic concerns.^2^ For aluminum and aluminum-based alloys, applications range from traditional structures such as aircraft components^3^ to more modern systems such as current collectors in battery devices.^4^ In particular, localized corrosion is one of the most difficult defects to detect because it can be submillimeter in size and be hidden by corrosion products.^5,6^ Yet these defects can penetrate deep into metallic parts or create sites of stress concentration that can lead to the initiation of fatigue cracks, overall degradation, and, ultimately, material failure.^7^ Frankel and Sridhar defined localized corrosion as a local breakdown of the passive film protecting the surface of the metal, resulting in accelerated dissolution of the metal beneath the passive film.^7^ When such an event occurs on an open surface, the phenomenon is known as pitting.^7^ Pitting corrosion is also characterized by the presence of aggressive anionic species in the surrounding environment, such as chloride, bromide, or iodide ions.^7–9^ Localized corrosion and pitting corrosion have been studied for decades, and a good understanding of the phenomenon has been achieved overall.^7,9,10^ Four main stages in the pitting corrosion have been distinguished,^11^ which are (i) the processes occurring on the passive film at the boundary between the passive film and the solution; (ii) the processes occurring within the passive film when no visible, microscopic changes are evident; (iii) the formation of so-called metastable pits that initiate and grow for a short period below the critical pitting potential and then repassivate, representing an intermediate step in pitting; (iv) stable pit growth above a certain potential, referred to as the critical pitting potential. Although numerous works have studied the latter two stages, little is known about the initial two. Therefore, the global mechanism by which localized corrosion proceeds remains elusive and is much debated.^10–12^

The challenge is to unambiguously characterize these early-stage phenomena, as they occur stochastically at the solid–liquid interface on a very small scale, with a passive film and initiation sites in the nanometer range.^13^ Traditionally, electrochemical techniques have been used to study corrosion processes and derive mechanistic insights,^10,14^ but they do not provide local information. X-ray photoelectron spectroscopy (XPS) combined with scanning tunnelling microscopy (STM)^15^ and X-ray absorption spectroscopy (XAS)^16^ have been implemented for the investigation of effects such as solution composition, applied potential, and the ion concentration on the formation of pits. More recently, scanning electrochemical cell microscopy (SECCM) has been used to study localized corrosion in polycrystalline Zn films,^17^ linking pitting initiation to grain boundaries. However, a number of technical issues remain, the main one being the instability of the exposed surfaces as a function of the reaction time. Additionally, techniques that enable the correlation of electrochemical measurements with additional real-time information have been shown to enhance our understanding of the dynamics of pit formation. In this regard, Frankel studied the growth of pits in Al thin films with thickness varying between 100 and 200 nm and recorded video images of the growth of the pits.^18^ By performing potentiodynamic controlled electrochemical measurements, it was demonstrated that pits in Al thin films quickly become two-dimensional and grow as radially increasing rings, in a manner similar to very small three-dimensional pits.^18^

Another technique that has shown great promise in providing insights into the local corrosion processes is liquid-phase electron microscopy (LPEM).^8,19^ This technique uses specialized holders designed to hermetically enclose liquid solutions sandwiched between two microelectromechanical systems (MEMS)-based chips.^8,20,21^ To allow for electron imaging, thin SiN_*x*_ membranes are suspended by locally etching the MEMS chips, typically revealing an imaging region on the order of 150 μm × 50 μm. One of the MEMS chips can be patterned with metallic electrodes to allow biasing through an external potentiostat. Therefore, electrochemical measurements can be conducted directly within the electron microscope while simultaneously collecting electron signals of the region of interest. Previous research on corrosion phenomena using LPEM focused on thin metallic film structures, where Chee et al. first reported on free corrosion (i.e., without external electrochemical stimuli) of Al electrodes using LPEM in transmission (LPTEM) mode. They investigated the effects of the concentration of NaCl solutions and the presence of Au^+^ ions in the system. Then, they carried out a morphological in situ and ex situ study again on free corrosion, showing the formation of a range of different structures, from blisters to round pits to fractal corrosion, when the Al films were immersed in NaCl solutions of different concentrations over an extended period of time.^22^ Recently, free corrosion on Fe films using LPTEM was also reported.^23^ In more relevant studies, potentiodynamic control for initiating pitting in Al films exposed to NaCl solutions was implemented,^24^ resulting in fractal networks only, while Pinkowitz et al. used a combination of linear sweep voltammetry and potentiostatic polarization to study Al pitting corrosion in 0.1 M Na_2_SO_4_ and 10^–3^–10^–5^ M NaCl solutions.^25^ They suggested that hydrated Al species in the active pit redeposit on uncorroded cathodic surfaces during localized corrosion. Importantly, they noted that pitting corrosion may occur preferentially in electron-beam-irradiated regions, suggesting technical challenges also associated with LPEM. While previous studies have demonstrated the high potential of LPEM in bringing additional insights into the early stages of pit formation, no work has reported on real-time information and its direct correlation with the electrochemical signal as yet.

Herein, we aim to address this gap using LPEM to image the formation and growth of electrochemically induced pits in real time. To achieve this, we immersed a thin film of metallic aluminum in a saline environment of 0.1 M NaCl, and we monitored the localized corrosion events in real time using LPEM. For full control of the metal and metal surface undergoing corrosion, we first microfabricated microchips containing a pure Al working electrode, which we characterized in terms of roughness, thickness, and chemical elements of the surface passivation film. We describe the experimental setup to achieve galvanostatic control from the beginning of the process, for which we performed chronopotentiometry measurements at different current values. By following the corrosive events on the Al working electrode using liquid-phase scanning electron microscopy (LPSEM), we show that the process starts with the formation of pits and the appearance of blisters, while at low current values, round and fractal-like morphologies coexist. At higher current levels, the morphology of the corrosion events exhibits only fractal-like characteristics, with the relative diameter of the fractals increasing with increasing current. Finally, at even higher current values, gas bubble formation is shown to occur in parallel to the pit growth, flooding the microcell. A closer look with LPTEM shows that gas generation also occurs at a lower applied anodic current at the front of the corrosion events, while electron energy loss spectroscopy (EELS) measurements confirm that the gas is molecular hydrogen. We also calculated oxidation rates from the experimental data and compared them with theoretical values. Finally, we conclude with a correlation of our observations with previous work to evidence the mechanism behind the localized corrosion of aluminum in the presence of Cl^–^ ions.

## Results

### Fabrication of Al Working Electrode on Microchips and Its Characterization

To study Al corrosion using LPEM, electrochemical top chips featuring a single Al working electrode (WE) were microfabricated in-house. The Al WE and two Pt electrodes (referred to as counter and reference electrodes, CE and RE) were lithographically patterned and e-beam evaporated on a Si wafer deposited with a 50 nm SiN_x_ layer, with the overall design shown in Figure 1a. Based on previous work,^26^ a circular symmetrical design was chosen for the geometry of the Pt electrodes in order to obtain a smooth current distribution during the experiments. The Al WE was designed with a “finger”-like configuration to allow preferential pitting sites at the top and edges of the fingers, as can be seen in the close-up SEM images in Figure 1b,c. The chips were locally etched to obtain a window where only a suspended, electron-transparent SiN_x_ membrane remained, as highlighted in Figures 1a–c and S1b,d. To concentrate the corrosion phenomena in the imaging region, a polymeric layer was spin-coated and lithographically patterned to define an electrochemically active region close to the imaging region. As highlighted in blue in Figure 1b,c, the polymeric passivation layer covers the Al WE up to the SiN_x_ membrane, leaving only the region of the Al WE fingers exposed to the electrolyte for imaging. We note that the Pt electrodes also have a thin Ti adhesion layer to avoid possible delamination during the electrochemical experiments, while no adhesion layer was used for the Al electrode due to galvanic coupling or side reactions with the underlying Ti that could cause serious interference with the system studied.^9^ Hence, by using an in-house-fabricated chip with a pure Al WE, we can well monitor the electrochemical performance of the system.

**Figure 1:**
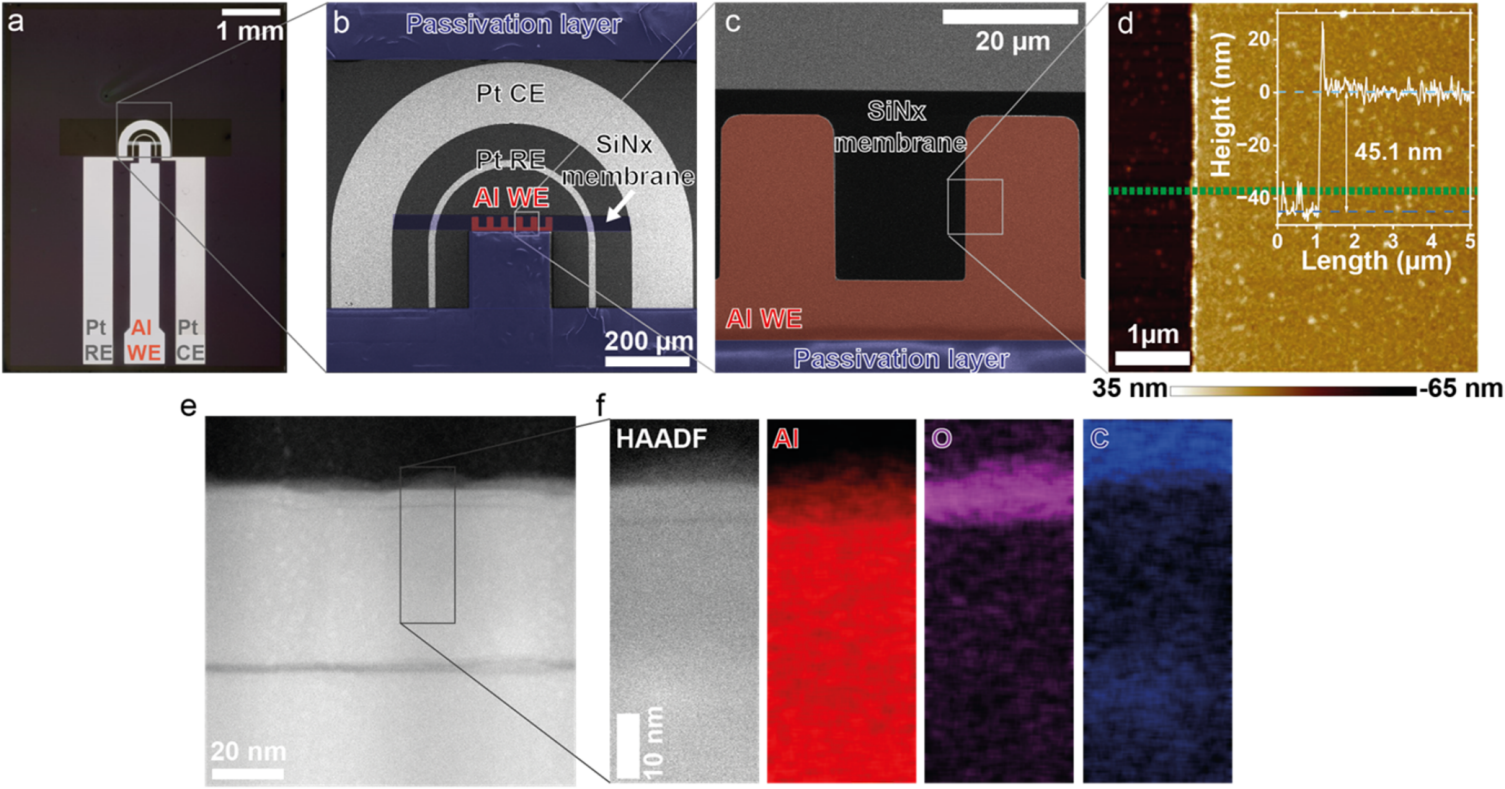
Characterization of the Al WE. (a) Optical microscopy image of an electrochemical LPEM top chip featuring one Al electrode and two Pt electrodes, a SiN_x_ membrane, and a polymeric passivation layer. (b, c) SEM images of the top chip depicting the symmetrical geometry of the two Pt electrodes with respect to the Al electrode and the finger-like shape of the Al electrode in the imaging region. (d) AFM image of the boundary between the SiN_x_ membrane and the Al electrode. The inset profile corresponds to the line scan performed on the green dashed line. (e) STEM HAADF image of a FIB lamella showing the cross section of the top chip. (f) STEM EDX measurement of the FIB lamella.

We first performed atomic force microscopy (AFM) measurements to quantify the thickness and roughness of the fabricated Al layer, Figure 1d. As shown in the profile inset of Figure 1d, the thickness of the Al layer is 45 nm, the average roughness *R*_a_ is 2.14 nm, and the root-mean-square average roughness *R*_q_ is 3.01 nm. Both values correspond to the expected roughness of e-beam evaporated Al thin films.^27^ The thickness of the Al layer was also confirmed by scanning transmission electron microscopy high-angle annular dark-field (STEM HAADF) imaging (Figure 1e) of a focused ion beam (FIB) prepared lamella from the cross section of a fabricated chip. STEM energy-dispersive X-ray spectroscopy (EDX) measurements (Figure 1f) on the same lamella revealed a native oxide thickness of 4–6 nm, typical of Al layers exposed to air.^28,29^ Additionally, TEM selective area diffraction (SAED) confirmed the polycrystalline microstructure of the patterned Al WE, as shown in Figure S2a.

### In Situ Galvanostatic Measurements in SEM Microcells

First, the custom electrochemical Al chip and a bottom spacer chip were assembled on a dedicated stage for electrochemical LPSEM experiments; see Figure S1a,b. The on-chip Al electrode was connected as WE, the most external on-chip Pt electrode as CE, and the remaining on-chip Pt electrode as RE, to form a 3-electrode electrochemical system. Details on the calibration of the Pt quasi-reference can be found in the Materials and Methods section and in Figure S3. The SEM configuration allows for imaging of the full electron-transparent window (Figure S4) and can provide an overview of corrosion events on all aluminum fingers simultaneously. The electrolyte, 0.1 M NaCl, was injected through the inlet after assembly, and static, full-cell liquid immersion conditions were used for secondary electron imaging, as schematically illustrated in Figure S1a,b. Control experiments confirmed that the system is stable under low-dose electron imaging (Note S1), while a custom denoising and data processing pipeline was developed to denoise and process the data; for details, see Methods and Figure S5.

Potentiodynamic measurements are typically used to monitor the corrosion processes in bulk systems. However, in the case of microcells, we have found that the processes induced by linear sweep voltammetry (LSV) stimuli proceed rapidly, leading to a loss of information on the early stages of the localized corrosion phenomena (Figure S6 and Video S1). This is due to the kinetics of the metal oxidation induced once the pitting potential is reached, which is too fast to be adequately recorded with the time resolution of a scanning electron microscope. Instead, we use galvanostatic control of the corrosion events, where slower kinetics can be achieved, allowing these early stages to be properly recorded and followed in real time, within the time resolution of electron microscopy measurements.

Chronopotentiometry (CP) measurements were performed at 1, 5, 10, 20, and 50 nA for a duration of 10 min. Figure 2 shows the electrochemical curves, representative SEM images at different time points, and post-mortem SEM images exhibiting the morphological evolution. The complete sequences are included in the Supporting Information (Figures S7–S11 and Videos S2, S3, S4, S5, and S6, respectively). For each measurement, the first 60 s correspond to the open-circuit voltage (OCV). For all the current values, the OCVs are relatively similar. They range from −1.08 to −1.35 V vs Ag/AgCl, and the maximum variation in a single OCV curve is 151 mV. This shows the relative reproducibility and stability of the chips used for these measurements.

**Figure 2:**
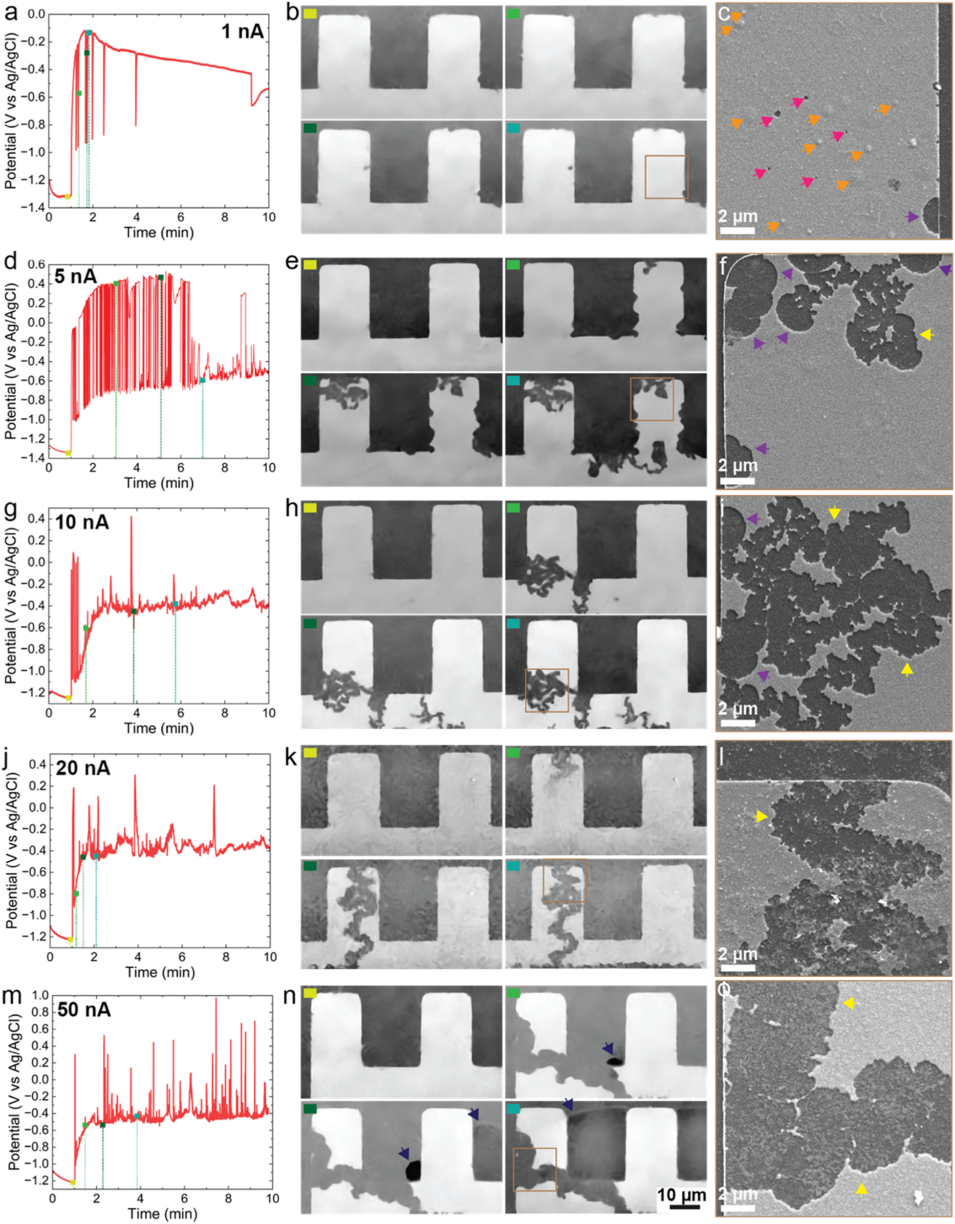
Time series of CP measurements performed at 1, 5, 10, 20, and 50 nA in the SEM. For each line, the electrochemical curves are depicted on the left, the middle panel corresponds to in situ acquired SEM images at different time points, followed by representative post-mortem SEM images shown on the right. The color of each top left square on the SEM images in (b, e, h, k, n) corresponds to a time point with the same color in (a, d, g, j, m), respectively. The regions highlighted with a brown square in (b, e, h, k, n) correspond to the post-mortem imaged regions in (c, f, i, l, o). In the post-mortem images (c, f, i, l, o), magenta arrows indicate pits, orange arrows indicate unruptured blisters , purple arrows indicate circular corrosion events, and yellow arrows indicate fractal patterns of the corrosion events. In (n), the dark blue arrows indicate the formation and expansion of a gas bubble.

At the start of the CP measurement, all curves begin with a sharp increase in the potential. This behavior was also observed in similar experiments performed within an ex situ setup using a bulk Ag/AgCl RE and a Pt wire as CE and, on the bench inside the LPSEM holder, as detailed in Note S2. This corresponds to a typical galvanostatic charging response, where this initial increase in potential is attributed to the pseudoohmic resistance and the subsequent charging of the interfacial capacitance. This process occurs over a period ranging from a few milliseconds to a few seconds, depending on the amplitude of the current density.^30–32^ In this work, the current densities of the CP measurements range from approximately 20 μA/cm^2^ to 1.2 mA/cm^2^ so that galvanostatic charging is expected to occur in the order of one to several seconds. After a maximum in the potential is reached, a sharp drop to a potential plateau of active anodic oxidation is usually observed.^30,32^ In the CP measurements shown in Figure 2, this plateau is preceded by a slow rise in potential, most likely due to the stabilization of the Pt quasi-reference used as RE. It was indeed shown that measurement performed using a bulk Ag/AgCl RE shows a direct transition from the galvanostatic peak to the oxidation plateau, without a transitory regime, as depicted in Note S2. Additionally, control experiments confirmed that the electron beam irradiation conditions used for the SEM imaging do not affect the measured electrochemical signal (more information is given in Note S1, while the calculation of the dose rate and total irradiation dose of these experiments is detailed in Note S3).

Figure 2a–c shows the measurements for the lowest applied current of 1 nA. At this current, the galvanostatic charging takes a few minutes (first downward peak), and the resulting increase in exposed area each time a pit forms is fast enough that the active current density in the pit quickly drops below the critical current density for pit growth. Thus, the repassivation of the formed pits is quasi-instantaneous. This implies that each downward peak in the electrochemical signal corresponds to the formation of a single pit, as seen in the SEM images in Figure 2b. These pits are metastable pits, corresponding to the third main stage of pitting corrosion.^11^ The post-mortem characterization, Figure 2c, reveals the presence of a greater number of initiated pits than those detected in situ (cf. magenta arrows in Figure 2c). It appears that only pits that reach the SiN_x_ substrate are detected electrochemically and visually by LPSEM. This may be because it is only the larger metastable pits that experience a variation in local current density that is large enough to be detected on the scale of the entire electrode. Monte Carlo simulations, detailed in Note S4, have shown that, even with reduced thickness, the majority of the electrons escaping the liquid cell originate from the Al WE until its complete corrosion. Figure 2c also shows the presence of small blisters on the WE (cf. orange arrows in Figure 2c), which were not detected at higher currents (cf. Figure 2f,i,l, and o). Blisters are known to form in the very early stages of pit formation when the chloride ions penetrate the oxide film and dissolution of the metallic aluminum occurs at the oxide–metal interface.^24,33^ It has been reported that hydrogen is produced when aluminum dissolves, creating a gas pocket between the metal and the oxide, known as a blister.^34–38^ The amount of hydrogen generated depends on the current density and, consequently, the applied anodic current. At low current densities, such as the measurement performed at 1 nA, the stress induced in the oxide film remains below the rupture value and enables post-mortem detection of blisters on the Al electrode.^24,33^ However, at larger applied anodic currents, the amount of hydrogen produced exceeds the rupture value, rupturing the blister and exposing the bare metallic aluminum to the electrolyte, thereby forming pits.^24,33^

At 5 nA, oscillations in the potential are recorded from the start of the CP measurement until 6.5 min into the experiment (Figure 2d). Similarly to the previous measurement performed at 1 nA, each downward peak of these oscillations corresponds to the formation of a metastable pit. However, this time, the process exhibits an accelerated rate due to the applied anodic current being five times larger. The initial shape of the corrosion events captured in the images follows a circular pattern before switching to fractal-shaped features after the fourth minute of the measurement (Figures 2e,f, S8 and Video S3). This transition is related to the use of galvanostatic conditions for measuring corrosion, as the applied current density increases with the degradation of the aluminum electrode during the measurement at constant current. Therefore, the rise in the applied current density during the measurement is reflected by a change in the shape, shifting from metastable pits (round-shaped) to more stable ones (fractal-shaped) that grow within the Al film. Finally, the flatter region of the electrochemical signal from 6.5 min into the measurement is attributed to corrosion progressing beneath the polymeric passivation layer, as revealed through post-mortem optical imaging depicted in Figure S14. This corrosion, which is possibly linked to crevice corrosion, is a side reaction caused by the configuration of our system. It is known that confined spaces, such as the geometry of our setup, can be preferential sites for localized corrosion due to an increased concentration of trapped chloride ions beneath the polymeric layer, causing a change in the local pH value.^9^ After this 6.5 min mark, the electrochemical signal transitions to a potential plateau, where no significant pitting events were detected in the imaged region. The minor degradation resulting from corrosion events that started under the passivation layer and progressed toward the Al electrode up to the imaged area observed after this timeline is also likely linked to crevice corrosion (Figure S8 and Video S3).

Increasing the applied current to 10 nA results in fractal-shaped corrosion events being imaged within the first minute of the galvanostatic measurement (Figures 2g,h, and S9, and Video S4). These events again correlate with oscillations in the electrochemical signal, indicating multiple pit formations during this stage of the experiment. The electrochemical signal then stabilizes, likely corrosion progressing under the passivation layer in a crevice corrosion regime analogous to the measurement performed at 5 nA. Similar corroded regions beneath the polymeric layer were visible in the post-mortem optical image of the electrochemical chip used for this measurement (Figure S14). Peaks in the potential at 3.5 and 5.5 min correspond to new pits forming in the imaged region, as indicated in Figure 2h.

At 20 nA, the electrochemical curve in Figure 2j shows that the first corrosion event occurs immediately after the initial galvanostatic charging. This corrosion event also shows a fractal pattern extending from the top of a finger of the Al WE to the polymeric passivation layer (Figure 2k). From 1 min 30 s to 2 min, corrosion occurs along the passivation layer and is reflected by several spikes in the electrochemical signal. A second corrosion event from the top of another Al finger occurs at 3 min 45 s, corresponding to the prominent peak detected in the electrochemical signal (see Video S5). The other prominent peak in the potential occurring at 7.5 min does not correspond to any change in the SEM images. It most likely corresponds to another corrosion event occurring under the passivation layer.

The CP measurement performed at 50 nA shows that the first corrosion event occurs immediately after the initial galvanostatic charging (Figure 2m,n), similar to the measurement performed at 20 nA. A broad fractal pattern is detected starting from one edge, and in contrast to other measurements, we directly image gas bubble nucleation and its subsequent growth. A second fractal-shaped pit starts at 3.5 min in the measurement, initiating from under the polymeric layer, and is associated with a spike in the potential (Video S6). The successive potential peaks detected at 4.5 min are also linked to the expansion of the corroded region. A second gas bubble formed at the end of this second corrosion event. The two gas bubbles merge at 5 min and cover most of the imaged area at 6 min. Several peaks in the electrochemical signal are also detected but do not correspond to any corrosion event happening on the imaged WE. Some of them are likely related to gas nucleation, expansion, or corrosion progressing beneath the passivation layer. The gas bubbles imaged are likely to be hydrogen, as previously reported.^18,33,39–42^

Image processing of the real-time SEM-acquired image sequences of the corrosion events enables the calculation of the oxidation rate at different applied anodic currents by isolating and determining the number of pixels removed by pitting corrosion for each frame of each experiment (see Materials and Methods and Figure S5). The oxidation rate is usually determined using Faraday’s law, according to the expression

1 $$r=\frac{i\cdot M}{n\cdot F}$$

where the corrosion rate, *r* (in g/(cm^2^·s)), is given as a function of the molar mass *M* of the oxidized metal (in g/mol, the molar mass of Al being *M*_Al_ = 26.98 g/mol), the applied current density *i* (in A/cm^2^), the number of electrons *n* involved in the reaction, and the Faraday constant *F* (96485 C/mol). It should be noted that the applied current densities used for determining the corrosion rate were obtained by dividing the applied current by the initial area of the working electrode. Therefore, the effective corrosion rate is expected to slightly increase with the duration of the CP measurement as the working electrode is degraded and its area decreases.

To calculate the effective corrosion rates from the LPSEM imaging experiments, we assumed a uniform thickness of the corroded Al electrode, which is a reasonable approximation given the conformal nature of e-beam evaporated electrodes. Then, the experimental corrosion rates *r*_exp_ were obtained by multiplying the measured surface removal rate *r*_LPEM_ (in cm^2^/s) by the thickness of the working electrode (*t*_WE_ = 5·10^–6^ cm) normalized to the density ρ of the oxidized metal (ρ_Al_ = 2.70 g/cm^3^) and the area of the working electrode (*A*_WE_ = 42.5·10^–6^ cm^2^), as shown in eq 2.

2 $$r_{\exp}=r_{\mathrm{LPEM}}\cdot t_{\mathrm{WE}}\cdot \rho_{\mathrm{Al}}\cdot \frac{1}{A_{\mathrm{WE}}}$$

Figure 3 shows the corrosion rates determined using eq 1, which follows Faraday’s law, and the experimental ones measured from the LPEM experiments performed in the SEM and calculated with eq 2 as a function of the anodic current applied to the CP. Both methods show a similar general trend, which highlight the reliability of the LPSEM measurements. At 5 nA, the experimental corrosion rate is lower than that predicted by Faraday’s law. This is probably because metastable pits mainly formed during this measurement, whereas Faraday’s law assumes continuous corrosion. Since the experimental process is slow and is not continuous, a lower experimental corrosion rate is to be expected. However, at 50 nA, the oxidation rate obtained experimentally is much higher than that determined using Faraday’s law. This could be related to the gas evolution observed during the experiment. Previous reports have indicated that the hydrogen evolution at active corrosion sites can considerably sustain and increase the local corrosion rate.^41^ For the intermediate currents of 10 and 20 nA, the experimentally obtained oxidation rates agree well with Faraday’s law within a 10% margin. At 10 nA, the lower corrosion rate calculated experimentally may be due to the formation of metastable pits at the start of the measurement. Additionally, it cannot be excluded that differences with the predictions of the Faraday law could also be caused by the different methods used to calculate the rates and/or hydrogen evolution reaction(s) and its contribution at varying currents (see Materials and Methods section for details on the surface removal rate determination).

**Figure 3:**
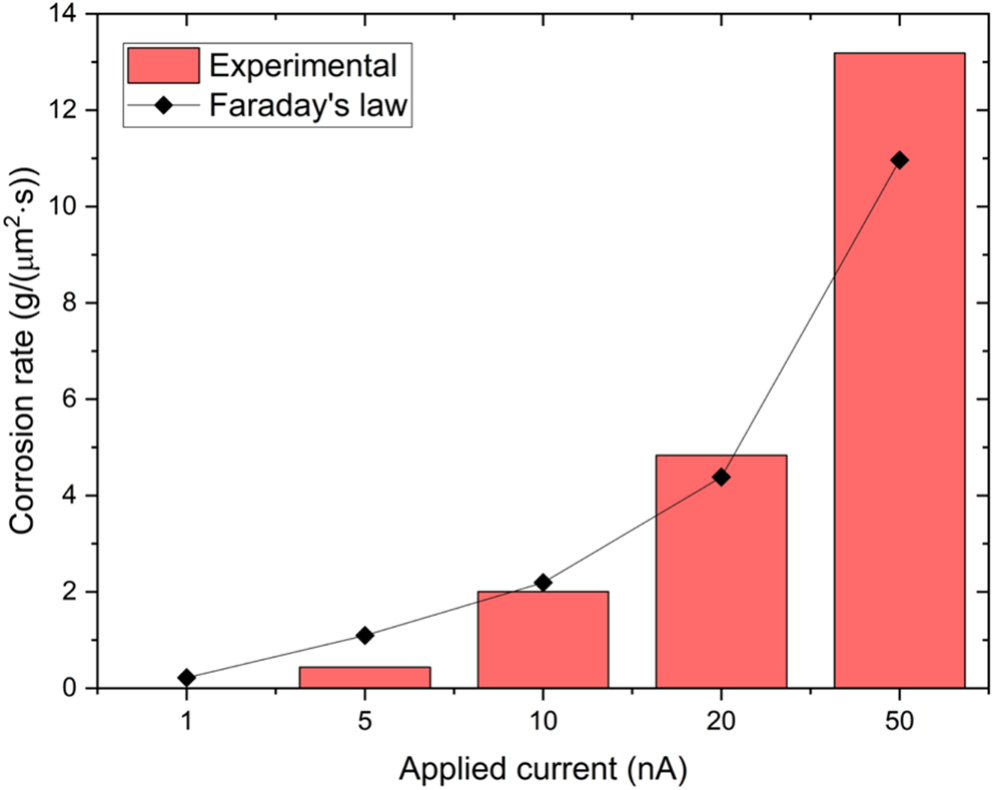
Al corrosion rates as a function of the applied current. The black diamond symbols depict the corrosion rates determined following Faraday’s law, whereas the red bars show the experimentally acquired corrosion rates.

### In Situ Galvanostatic Measurements in TEM Microcells

The microcell was also mounted on a dedicated stage for galvanostatic LPTEM measurements (Figure S1c,d), with the 0.1 M NaCl electrolyte injected through the inlet after assembly to obtain static thin liquid layer immersion conditions. Figure 4 depicts the CP measurement at 5 nA and representative TEM images at different time points. The full sequence is included in the Supporting Information, Video S7. Similar to the SEM measurements, the first 60 s correspond to the OCV acquisition, and the CP measurement shows a galvanostatic charging peak followed by a potential plateau of active anodic oxidation. This time, however, the OCV values range between −0.99 and −0.84 V vs Ag/AgCl, which are shifted compared to those obtained in the SEM. Furthermore, the oscillations in the potential detected immediately after the galvanostatic charging during the LPSEM experiment, which was performed at the same applied anodic current (Figure 2d), are not observed in the LPTEM electrochemical signal (Figure 4a). The peaks observed between 1.5 and 2.0 min in the LPTEM measurement do not correlate with any imaged corrosion event and likely correspond to corrosion occurring at another location on the exposed aluminum electrode or progressing beneath the passivation layer. In contrast to the LPSEM measurement at the same applied current, the imaged corrosion events in the LPTEM measurement display a fractal pattern from the beginning of the CP measurement. This corrosion behavior is more similar to what is observed at applied anodic currents of 10 or 20 nA in the LPSEM experiments. These discrepancies are indicative of the influence of electron-beam-induced effects at the scale of TEM imaging, which are more pronounced than during SEM imaging. This could be explained by the considerable difference in the dose rate used between SEM and TEM acquisition, where the TEM dose rate for this experiment was estimated to be 1000 times higher than the SEM ones (more details can be found in Note S3). Additionally, the thin liquid configuration used in the TEM, as illustrated in Figure S1d, could also impact the system’s dose-related tolerances potentially influencing the diffusion of the radiolytic species and consequently the local pH value. Despite the clear difference in the kinetic behavior of the system in TEM, the progression of corrosion on a single Al finger is clear. Interestingly, the images reveal that the corrosion front exhibits brighter contrast (Figure 4b, yellow arrows). We hypothesize that this contrast is due to gas being produced when metallic Al surfaces are newly exposed to the aqueous electrolyte. Previous reports suggest that this gas is hydrogen.^18,33,39–42^ According to Wiersma and Hebert,^38^ molecular hydrogen could result from a parallel oxidation process occurring during anodic pit dissolution, following the chemical reaction shown in eq 3. The fact that such a small amount of gas can be imaged suggests that it may be trapped beneath the remaining oxide layer before diffusing into the electrolyte. Therefore, it is probable that some hydrogen generation contributes to the mechanical removal of the oxide layer during pit growth.

3 $$\mathrm{Al}+2H_{2}O\to \mathrm{AlOOH}+\frac{3}{2}H_{2}$$

**Figure 4:**
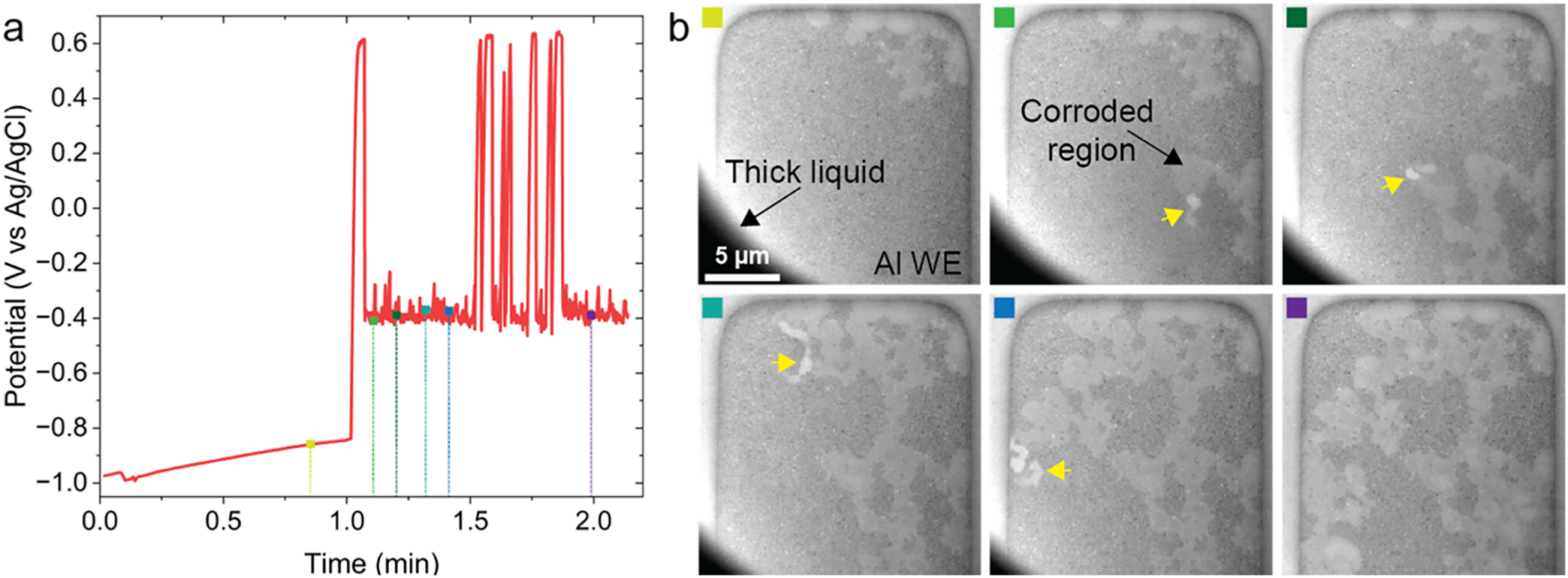
Time series of the CP measurement performed at 5 nA in the TEM. (a) Electrochemical curve. (b) TEM images acquired at a dose rate of 0.64 e^–^·nm^–2^·s^–1^. The color of each upper left square in the TEM images in (b) corresponds to a time point with the same color in (a). For each TEM image, the Al WE corresponds to the region with the darker salt and pepper signal, whereas the corroded region corresponds to the brighter fractal-like region. The black area in the bottom left corner of the TEM images corresponds to a thick liquid layer. The yellow arrows indicate brighter contrast at the corrosion front attributted to the presence of gas.

To confirm the chemical fingerprint of the gas that is formed at the corrosion front, we induced an electrochemically formed gas bubble at 50 nA using CP and acquired STEM EELS. Details of the experiment are given in Note S5. Figure 5a shows a representative annular dark-field (ADF) STEM image. The bright contrast corresponds to the liquid surrounding the electrochemically formed gas bubble, which shows higher transparency due to lower electron scattering in gases than in liquids. EELS measurements were performed on the Al electrode and the SiN_x_ membrane (Al free) in the gas-filled region and compared with the corresponding spectra obtained in a vacuum, Figure 5b. The low-loss EEL spectra on the Al electrode are dominated by the presence of the very sharp plasmonic peak of Al, with a maximum at around 15 eV associated with the Al volume plasmon.^43^ The high intensity of this peak does not allow for the evaluation of possible hydrogen production, whose ionization edge is at 12.5 eV, as reported by Crozier and Chenna.^44^ However, the EEL spectrum of the membrane region (also filled with the electrochemically induced gas) shows a fingerprint feature at 12.5 eV, confirming the production of molecular hydrogen during anodic corrosion.

**Figure 5:**
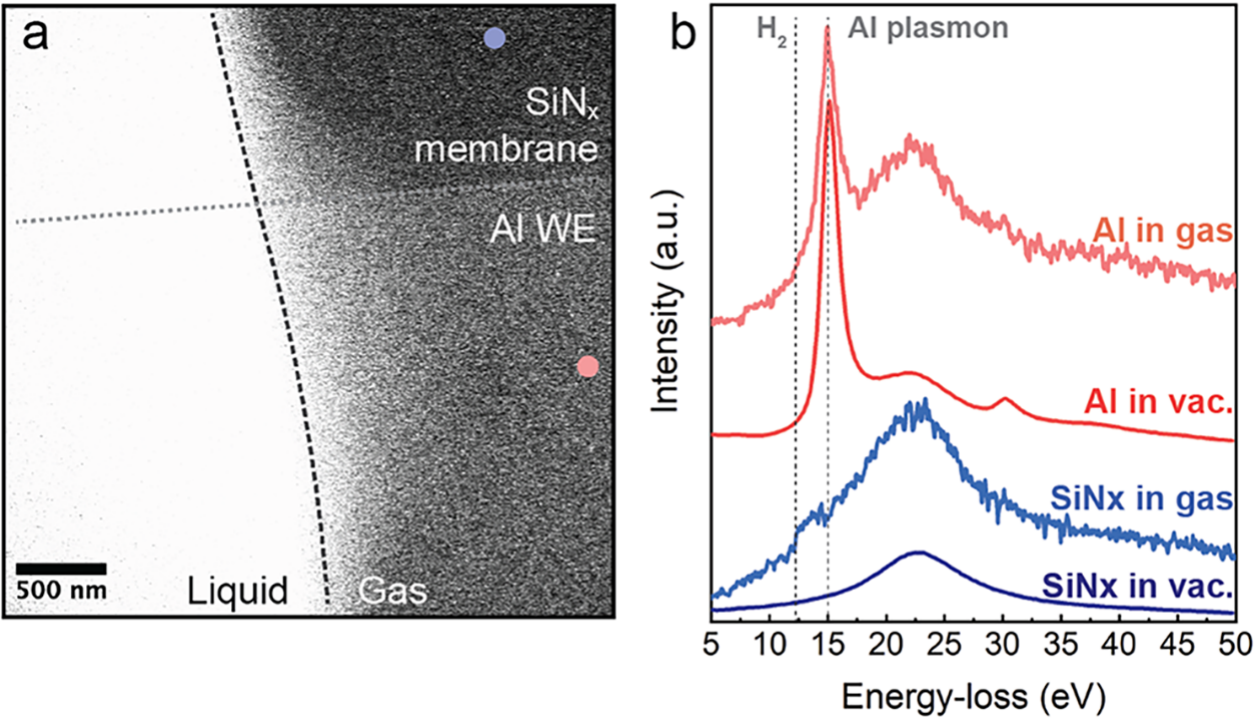
ADF-STEM image and EEL spectra of an electrochemically induced gas bubble using CP at 50 nA. (a) ADF image of the region used for EELS acquisition. (b) EEL spectra acquired in the microcell under vacuum (less noisy curves) and with the electrochemically induced gas bubble (noisy spectra). The colored spots highlighted in (a) correspond to the Al and SiNx EEL spectra acquired in gas in (b).

## Discussion

Previous studies^24,33^ have reported that localized corrosion is initiated when the Al electrode is immersed in the chlorinated aqueous electrolyte, where Cl^–^ ions adsorb and penetrate the native oxide until it breaks down. The exact form and mechanism of this breakdown remain uncertain^33^ although it is generally accepted that the metallic Al comes into contact with the chlorinated solution through the formation of a primary crack or pore in its native oxide layer.^45^ The increased roughness of the oxide surface of the post-mortem AFM-imaged LPSEM-corroded chips may indicate the presence of nanocracks (Figure S16). This proposed pit initiation mechanism cannot be corroborated by our real-time measurements. However, our results provide insight into the steps that follow pit initiation in the localized anodic Al corrosion mechanism, including blister formation and the formation of stable and metastable pits as well as the production of molecular hydrogen. Figure 6 illustrates the stages of pitting corrosion that were unambiguously observed and are discussed next in the context of previous studies.

**Figure 6:**
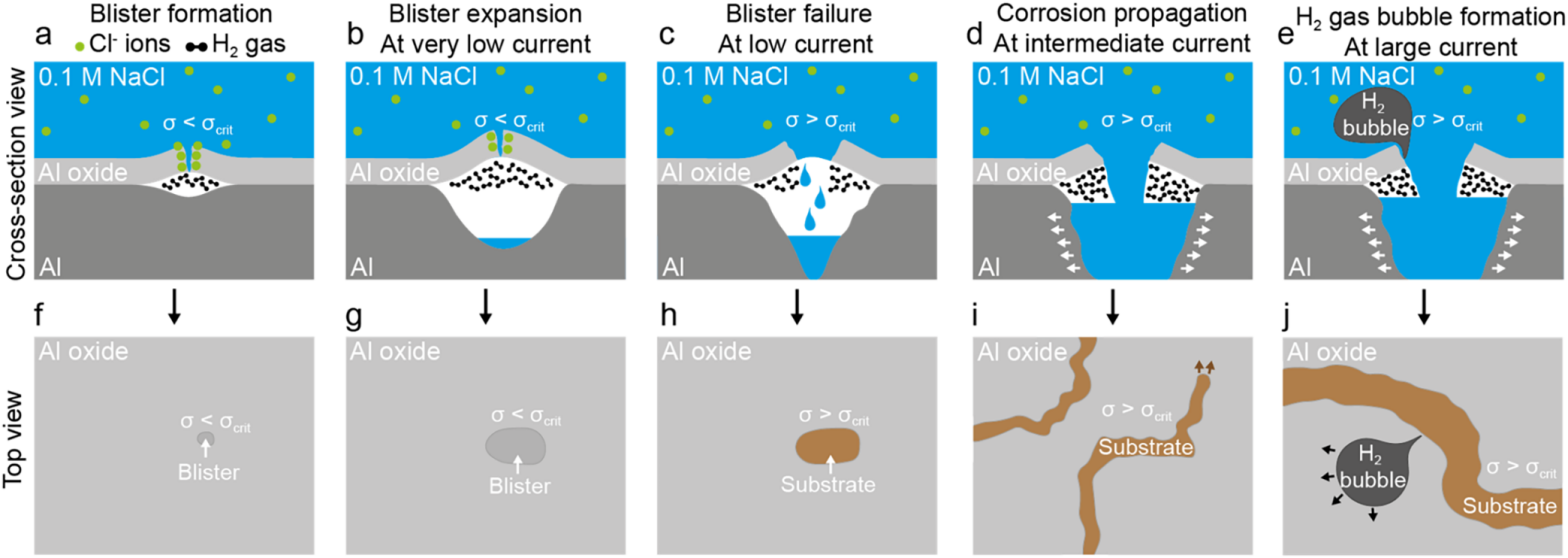
Schematic illustrating the observed steps of localized anodic corrosion of metallic Al passivated with the native oxide film. (a–e) Stepwise cross-sectional view and (f-j) corresponding surface view of the processes.

### Blister Formation

When metallic Al is exposed to the electrolyte, the process of metal dissolution begins. Several hypotheses have been proposed to explain the rapid initial dissolution of metallic Al, one being that its surface is initially unfilmed but later acquires a salt or adsorbed film that slows down the corrosion rate.^46^ Another proposed explanation is that the initial rapid corrosion rate is linked to an additional, parallel process to that of the anodic dissolution.^38^ This reaction could involve the oxidation of the metal directly under the oxide film by the water incorporated in the aluminum oxide, as described by eq 3, generating hydrogen at the metal–oxide interface.^38^ The complete chemical reaction pathway in chloride solution is complex and still under discussion; more details can be found elsewhere.^33,47^

The generated hydrogen causes the oxide layer to delaminate from the Al metal.^24,33,45^ This hydrogen pocket takes the form of a blister (Figure 6a,f), and the pressure within it increases as the corrosion reaction continues, inducing stress in the oxide layer. We believe that the stress generated by the formed hydrogen assists in the mechanical delamination of the oxide layer from the metal, contributing to the growth of the blister. The corrosion rate, and consequently the rate at which hydrogen is formed, is linked to the applied current density. In the framework of this study, this refers to the applied anodic current. When the applied anodic current is very low, hydrogen production is slow. Thus, the delamination of the oxide from the underlying metal is fast enough to keep the stress in the oxide layer below the rupture value (σ < σ_crit_), enabling the blister to continue growing (Figure 6b,g). The critical stress (σ_crit_) and the pressure required to rupture a blister can be determined using expressions previously derived by Ryan and McCafferty.^45^ This situation corresponds to the blisters detected in the post-mortem imaging of the LPSEM CP experiment performed at 1 nA (Figure 2c). The difference in size of the pits and blisters in that panel also highlights possible blister growth, linked to the increasing amount of hydrogen produced. As the applied anodic current is slowly increased, the rate at which molecular hydrogen is generated becomes high enough that the growth of the blister, which is linked with oxide-metal delamination, cannot compensate for the increase in the stress in the oxide layer. Consequently, the critical stress is reached and the blister ruptures (σ > σ_crit_; Figure 6c).

### Formation of Metastable and Stable Pits

The metallic Al is then fully exposed to the electrolyte (Figure 6h). However, corrosion does not progress further, as the rapid increase in the exposed area causes the active current density in the pit to drop below the critical current density required for pit growth. Therefore, it seems that the repassivation of Al metal in the formed pits is not only related to a repassivation potential, as previously suggested,^7^ but is also dependent on the active current density within the pit. Consequently, there is a range of current densities large enough to provoke blister rupture but low enough to enable pit growth. These pits are likely to correspond to those with a round shape detected in the LPSEM CP experiment performed at 1 nA (Figure 2b,c) and at 5 nA (Figure 2e,f). The round shape is thus attributed to the pit retaining the circular form of the ruptured blister. These nongrowing pits also fit the description of the metastable pits, as reported by Szklarska-Smialowska.^11^ At the intermediate applied anodic current, the blister ruptures, and the current density in the active pits is sufficient to enable pit expansion in the metallic Al electrode (Figure 6d,i). This mechanism corresponds to the fractal-shaped pits detected in the LPSEM CP experiment performed at 5, 10, and 20 nA (Figure 2e,f,h,i,k,l) and in the LPTEM CP experiment performed at 5 nA (Figure 4b). These pits exhibit stable growth, as described previously in Szklarska-Smialowska’s work.^11^ The TEM experiment suggests that hydrogen production may facilitate pit expansion by mechanically delaminating and removing the oxide layer at the corrosion front while simultaneously dissolving the underlying metal. It is unclear whether the crystallographic orientation of metallic Al plays a role in the direction of pit expansion. Hebert and Alkire^48,49^ and consecutive studies^50–52^ have reported on the preferential growth of Al tunnels in the ⟨100⟩ direction of high-purity Al foil in aqueous chloride solution at temperatures above 60 °C. However, no preferential orientation seems to prevail herein, as the pit growth follows a stochastic manner. This is likely due to the polycrystalline nature of the microfabricated Al electrodes, the structure of which remains unchanged after corrosion, as shown in Figure S2.

Our findings show that the change in the morphology of the corrosion events is related to the active anodic current density, which facilitates or impedes the progression of pit growth. Balázs and Gouyet previously reported that both corroded structure morphologies depend on the concentration of dissolved ions under free corrosion conditions.^53^ However, kinetics also plays a role in changing the corrosion pattern. The LPSEM CP experiment performed at 5 nA (or 0.1 mA/cm^2^ current density) shows that this value is close to the threshold at which the applied anodic current density is large enough to compensate for the rapid increase in the area of metallic Al exposed, thereby maintaining the active current density greater than the critical current density for pit growth. In this measurement, a transition occurs between round-shaped and fractal-shaped corrosion events, as the WE degrades and the electrochemically active area decreases. This, consequently, increases the current density until the transition threshold is reached. This value is thus close to that which allows for the change in corrosion morphology (Figure 2d–f). This current density value may also be referred to as a threshold for the transition between metastable and stable pits, as previously described in the literature.^11^

### Hydrogen Formation in the Framework of Pitting Corrosion

Finally, the LPSEM CP measurement performed at 50 nA (Figure 2n,o), which corresponds to the largest anodic applied current used, revealed the formation of large hydrogen bubbles alongside Al corrosion, as schematically illustrated in Figure 6e,j. The simultaneous occurrence of hydrogen evolution and Al pitting corrosion has been widely reported^18,33,39–42^ and is experimentally confirmed herein. Its detection in the LPSEM imaging could be due to hydrogen reaching its saturation limit within the electrolyte, leading to the formation of the detected bubbles. However, it is more likely that hydrogen evolves close to the active corrosion sites as a local cathodic reaction.^40^ As Al oxidizes, other species in the surrounding environment are reduced to maintain charge neutrality.^41^ For noble materials, the cathodic reaction typically involves the reduction of oxygen dissolved in the aqueous electrolyte.^41^ However, for materials with a low electrochemical potential, such as Al, it is thermodynamically possible to reduce hydrogen.^41^ In fact, when the electrolyte comes into contact with the metal, hydrogen can evolve due to a large potential difference available.^40^ Its occurrence at active corrosion sites, such as the pits formed, can considerably increase the local corrosion rate,^41^ and could explain the high corrosion rates obtained experimentally in Figure 3, or provide additional current to sustain corrosion propagation.^41^ Conversely, it has been reported that hydrogen evolution at active corrosion sites increases during anodic polarization.^40^ This increase in the rate of hydrogen evolution during anodic polarization is referred to as “superfluous hydrogen evolution.” ^40,41^ This phenomenon may cause large hydrogen bubbles to form during anodic Al corrosion at a high applied current.

However, the hydrogen bubbles observed at 50 nA are much larger than those generated during blister formation and pit expansion, as revealed by the LPSEM measurement performed at 1 nA (Figure 2a,b) and the LPTEM measurement performed at 5 nA (Figure 4). Additionally, the positions of small and large hydrogen bubble formations do not coincide. The large hydrogen bubbles (at 50 nA, Figure 2m–o) originate from the Al electrode and are not directly linked to pits, as they continue to grow even when there is no evidence of active pit growth in their immediate vicinity. On the other hand, the tiny hydrogen bubbles (observed in TEM at 5 nA, Figure 4) are located directly at the corrosion front. These discrepancies emphasize the possibility of multiple hydrogen sources in Al pitting corrosion. The local nature of the tiny hydrogen bubbles suggests a mechanism in which Al dissolution would directly lead to hydrogen formation. In this regard, the mechanism proposed by Wiersma and Hebert,^38^ following the relationship shown in eq 3, suggests that a small amount of hydrogen is also formed directly at the metal–oxide interface, as clearly indicated by the LPTEM observations of the propagation of the corrosion front.

## Conclusions

In conclusion, we have developed an experimental platform for the real-time and in situ study of localized anodic corrosion using electrochemical LPEM and have implemented it on pure Al films. By performing in situ galvanostatic measurements in the SEM at different applied currents, we observed different corrosion mechanisms, ranging from the detection of single pits and blisters to the observation of round- and fractal-shaped corrosion events and the simultaneous formation of gas bubbles. These mechanisms depend on the applied current and, therefore, on the corrosion kinetics. Calculation of the corrosion rates showed that the in situ processes follow the theoretical predictions at intermediate current values. In situ galvanostatic TEM measurements indicated the formation of gas at the corrosion front, which was experimentally confirmed to be molecular hydrogen. On the basis of these results, the current density threshold for the transition between metastable and stable pits in this polycrystalline Al thin film configuration was proposed, found to be approximately at 0.1 mA/cm^2^, while the production of molecular hydrogen was discussed in terms of its origins with respect to the currents applied. Our experimental platform can also extend beyond single metal corrosion, while its combination with the recently developed deep learning models^54^ can help identify the parameters that cause severe material degradation and propose descriptors for corrosion-resistant metallic systems.

## Materials and Methods

### Microfabrication of Al Chips

Top chips were microfabricated from a double-sided polished 200 μm thick Si wafer covered on both sides with 50 nm low-stress silicon nitride (SiN_*x*_) using low-pressure chemical vapor deposition (LPCVD, Centrotherm furnaces). To form the electron-transparent membranes, SiN_*x*_ was photolithographically patterned (1.2 μm thick AZ ECI 3007 positive tone resist; Süss MicroTec MA6 mask aligner) and etched using reactive ion etching (RIE, SPTS APS dry etcher) on its backside. Si was consecutively etched by wet etching in 20% potassium hydroxide (20% KOH, 80 °C, 2h 15 min), using the patterned SiN_*x*_ as a hard mask.

The electrodes were obtained using double-layer lift-off. First, they were photolithographically patterned (0.4 μm LOR/1.1 μm AZ 1512 HS, Heidelberg Instruments MLA140 laser writer) and deposited using e-beam evaporation (Leybold Optics LAB 600H evaporator at a deposition rate of 4.0 Å/s with a base pressure of 1.8·10^–6^ mbar). The excess of evaporated metal and photoresist was then lifted off by immersing the wafer in a photoresist stripping solution (Remover 1165, MICROPOSIT) for 24 h. The Pt electrodes were set up to be 50 nm thick and supported on a 5 nm thick Ti adhesion layer. The Al electrode was set up to be 50 nm thick without any adhesion layer.

A 800 nm thick polymeric (SU8 2000.5, Kayaku Advanced Materials) passivation layer was spin-coated (Sawatech LSM-250) and lithographically patterned (Süss MicroTec MA6Gen3) to define the electrochemically active region. The passivation was designed to cover the Al WE up to the SiN_*x*_ membrane to concentrate the pitting events in the imaging region.

Once these processes were done at the wafer scale, the wafer was diced into chips (Disco DAD321). A protective polymeric layer (5.0 μm AZ 10XT-60) was previously spin-coated to protect the chip during dicing. The protective layer was removed by immersing the Al chip for 30 s in acetone (≥99.8%, Analytical reagent grade, Fischer Scientific) and 30 s in ethanol (≥99.8%, Analytical reagent grade, Fischer Scientific) before chip usage.

### AFM and TEM Characterization

The produced electrochemical Al chips were imaged using an optical microscope and a Quattro environmental scanning electron microscope (ESEM, Thermo Fischer Scientific). The thickness and the roughness of the microfabricated Al electrodes were determined by performing AFM measurements (Bruker FastScan AFM) on a 5 × 5 μm^2^ area at the edge between an Al finger and the SiN_*x*_ membrane. Post-mortem AFM measurements were also performed on the LPSEM-corroded chips on a 10 × 10 μm^2^ area, at the edge of a pit, as well as on the remaining Al electrode. The average roughness $R_{a}=\frac{1}{l}\int_{0}^{l}\left|z\left(x\right)\right|dx$ and the root-mean-square average roughness $R_{q}=\sqrt{\frac{1}{l}\int_{0}^{l}z{\left(x\right)}^{2}dx}$ were calculated with *l* being the evaluation length and the profile height function as a function of the position *x*.

To characterize the native Al oxide thickness, a cross-section lamella of a chip was prepared using FIB milling (Helios G4 PFIB UXe) and imaged in STEM mode in a C_s_ double-corrected Titan Themis TEM (Thermo Fischer Scientific) operated at 300 kV and 100 pA probe current, with a convergence semiangle of 20 mrad and a camera length of 240 mm.

### Liquid Cell Assembly and Sample Preparation

For both in situ SEM and TEM experiments, the electrochemical liquid cell system is made of the Al top chip and a 2 μm spacer chip (Hummingbird Scientific). The spacer was air-plasma treated (HPT-100 Henniker Plasma) for 90 s at 50 sccm and 100% power to enhance its wettability before the assembly of the chips on the different holders. Prior to the SEM experiments, a 5 nm thick carbon coating was sputtered on the top chip backside using a carbon coater (Cressington Scientific Instruments) to mitigate the charging effect of the polymeric passivation layer. The cell was assembled on a bulk liquid electrochemistry SEM stage (Hummingbird Scientific Inc.) or a liquid electrochemistry TEM holder (Hummingbird Scientific Inc.) for the TEM ones. Once assembled, the cell was filled with a 0.1 M NaCl (Roth AG) aqueous electrolyte. The electrolyte was flown in the liquid cell through the inlet tube using a syringe until the liquid poured out the outlet tube (see Figure S1a,c). For the measurements performed in the SEM, the cell was completely saturated with liquid, whereas for the one performed in transmission mode, a thin liquid layer was used (Figure S1b,d). The filling of the liquid was confirmed under an optical microscope and from the stabilization of the open-circuit potential around a relevant value.

### Electrochemical Measurements

The electrochemical measurements were performed by using a potentiostat (Bio-Logic SP-200 and SP-300) with an ultralow current probe. The electrochemical system was a coplanar 3-electrode system using the electrochemical top chip electrodes: the Al one as WE and the two Pt ones as CE and RE, respectively (see Figure 1a,b). The Pt quasi-RE was calibrated relative to an Ag/AgCl reference potential, showing a stable potential around −0.22 V vs Ag/AgCl, as detailed in Figure S3, so that all electrochemical measurements are presented with respect to this potential. The aqueous electrolyte remained static during the electrochemical measurements.

To study Al corrosion, both free corrosion, potentiodynamic, and galvanostatic measurements are possible.^8,9^ In this work, potentiodynamic and galvanostatic methods were used. In the SEM, LSV was first performed from −0.1 V versus the OCV to +1.5 V versus the OCV at a 1 mV/s scan rate. Then, CP measurements were performed at 1, 5, 10, 20, and 50 nA for 10 min in the SEM and at 5 and 50 nA in the TEM. Both LSV and CP measurements were preceded by a 1 min OCV measurement to ensure the stability of the electrochemical system.

### Electron Imaging

The electrochemical LPEM experiments were performed in a Quattro ESEM instrument (Thermo Fischer Scientific) and in a Talos F200S TEM instrument (Thermo Fischer Scientific). In situ SEM recordings were acquired at 5 kV with a current probe of 64 pA and a dwell time of 1 μs using a secondary electron Everhart–Thornley detector. In situ TEM recordings were acquired at 200 kV with a current probe of 100 pA. Post-mortem SEM characterization was performed in a Quattro ESEM (Thermo Fischer Scientific).

STEM ADF and STEM EELS measurements were performed in a C_s_ double-corrected Titan Themis transmission electron microscope (Thermo Fischer Scientific) equipped with a Gatan GIF continuum HR EELS spectrometer and a Gatan K3 camera. The STEM ADF and EELS measurements were acquired with a 300 kV and 150 pA current probe. For acquisition in vacuum, a 20 mrad convergence semiangle and a 44.32 mrad collection semiangle were used with a 180 meV/channel dispersion. For liquid cell acquisition, a 20 mrad convergence semiangle and a 94.6 mrad collection semiangle were used with 90 meV/channel dispersion. EELS spectra were normalized such that the maximum intensity of their zero loss peak is equal for each spectrum.

Energy-filtered TEM SAED measurements were performed in a JEOL 2200FS instrument featuring an in-column Ω-filter. The SAED measurements were acquired at 200 kV, using a 4k × 4k Gatan OneView CMOS camera, a 10 eV energy filter, and a camera length of 800 mm. Radial profiles were normalized to have their higher intensity equal to 1 and were compared to reference diffraction planes of Al obtained by Mulder et al.^55^

### Image Processing

The acquisition of SEM images under low-dose conditions reduces the signal-to-noise ratio in the recorded images, making their direct analysis difficult (Figure S5b), which severely hinders image segmentation. Hence, the SEM images were denoised and segmented using a specific image processing pipeline depicted in Figure S5a. This pipeline was partly inspired by the work of Marchello et al., in which they presented an analysis pipeline for restoring images of soft organic materials acquired with LPEM.^56^ To denoise their images, they proposed first to apply a median filter and then the Progressive Image Denoising (PID) algorithm developed by Knaus and Zwicker.^57^ The latter combines both transform and variational denoising techniques to reduce noise in images and is relatively short compared to other state-of-the-art denoising methods.^57^

Thus, the image processing pipeline developed for this work first applied a 3D Gaussian blur to the trimmed SEM images (σ = 1 in the *X*, *Y*, and *Z* directions). Then, a median filter and the PID algorithm were applied, similarly to Marchello’s work.^56^ The obtained denoised images were then segmented using *Binarize* function in Mathematica software.^58^ This function can automatically binarize images using Otsu’s cluster variance maximization method.^59^ The segmented images allowed the number of pixels forming the Al electrode to be counted for each recorded image. The evolution of this number of pixels over time allows the kinetics of Al oxidation and corrosion to be determined. The image processing pipeline is schematically presented in Figure S5a, with an image corresponding to each step presented in Figure S5b–f.

It should be noted that depending on the applied anodic current, there are some discrepancies in the way the surface removal rate, *r*_LPEM_, is determined and its phenomenological significance. In the case of the LPSEM measurements performed at the higher currents of 20 and 50 nA, the removed area corresponded to a single corrosion event. In these cases, only the initial pit was used to determine the surface removal rate, not the subsequent one. This enables keeping consistency and ensuring an effective current density as close to the initial one as possible. In the case of the LPSEM experiments performed at 5 and 10 nA, multiple pits were growing at the same time on different locations of the working electrode. Thus, the surface removal rate could not be attributed to a single pit and was determined by measuring the reduction in the working electrode surface as a whole. Finally, for the smallest current of 1 nA, the changes in the working electrode area induced by the small corroded regions could not be distinguished from the changes in the intensity of the images within the measurement. Therefore, no experimental value was reported for the corrosion rate of the LPSEM measurement performed at this current.

## Supplementary Material

















## Notes

Liquid cell setups in SEM and TEM; TEM SAED analysis of microfabricated and corroded electrochemical chips; quasi-reference Pt electrode calibration; denoising and segmentation pipeline; potentiodynamic in situ Al corrosion in the SEM; corrosion events on the full imaged window for CP at 1, 5, 10, 20, and 50 nA; post-mortem optical images of LPSEM-corroded electrochemical chips; AFM post-mortem characterization of the LPSEM-corroded chips (Figures S1–S16); LPSEM control experiments of beam-induced degradation; reliability of the in situ galvanostatic measurements; electron dose calculation; Monte Carlo simulation of e-beam-irradiated liquid cells; in situ TEM CP and consecutive EELS measurement for molecular hydrogen detection (Notes S1–S5) (PDF)
Operando ec-LPSEM movie of the LSV measurement (Video S1) (MOV)
Operando ec-LPSEM movie of the CP measurement performed at 1 nA, corresponding to Figure 2a–c (Video S2) (MOV)
Operando ec-LPSEM movie of the CP measurement performed at 5 nA, corresponding to Figure 2d–f (Video S3) (MOV)
Operando ec-LPSEM movie of the CP measurement performed at 10 nA, corresponding to Figure 2g–i (Video S4) (MOV)
Operando ec-LPSEM movie of the CP measurement performed at 20 nA, corresponding to Figure 2j–l (Video S5) (MOV)
Operando ec-LPSEM movie of the CP measurement performed at 50 nA, corresponding to Figure 2m–o (Video S6) (MOV)
Operando ec-LPTEM movie of the CP measurement performed at 5 nA, corresponding to Figure 4 (Video S7) (MOV)
