# Supplementary material for: Pit Morphology, Dissolution Kinetics, and Gas Generation Monitored in Real Time during Localized Anodic Aluminum Corrosion
Source: J Am Chem Soc. 2025 Sep 10;147(38):34920–32. doi: 10.1021/jacs.5c11352 (PMC12464990; doi:10.1021/jacs.5c11352)
Supplement: Supplementary file 1 [file ja5c11352_si_001.pdf]

Supporting Information

Pit Morphology, Dissolution Kinetics, and Gas Generation Monitored in Real-Time during Localized Anodic Aluminum Corrosion

Morgan Barbey-Binggeli and Vasiliki Tileli*

[1]Institute of Materials, École Polytechnique Fédérale de Lausanne, CH–1015 Lausanne, Switzerland

*corresponding author: vasiliki.tileli@epfl.ch

Table of Contents

Supplementary Notes and Figures 2

Figure S1. Liquid cell setups in SEM and TEM 2

Figure S2. TEM selective area diffraction analysis of microfabricated and corroded electrochemical chips 3

Figure S3. Quasi-reference Pt electrode calibration 4

Note S1. LPSEM control experiments of beam-induced degradation 5

Figure S5. Denoising and segmentation pipeline 6

Figure S6. Potentiodynamic in situ Al corrosion in the SEM 7

Figure S7. Corrosion events on the full imaged window for CP at 1 nA 8

Figure S8. Corrosion events on the full imaged window for CP at 5 nA 9

Figure S9. Corrosion events on the full imaged window for CP at 10 nA 10

Figure S10. Corrosion events on the full imaged window for CP at 20 nA 11

Figure S11. Corrosion events on the full imaged window for CP at 50 nA 12

Note S2. Reliability of the in situ galvanostatic measurements 13

Note S3. Electron dose calculation 15

Note S4. Monte-Carlo simulation of e-beam irradiated liquid cells 16

Figure S14. Post-mortem optical images of LPSEM corroded electrochemical chips 17

Note S5. In situ TEM CP and consecutive EELS measurement for molecular hydrogen detection 18

Figure S16. AFM post-mortem characterization of the LPSEM-corroded chips 19

References 20

Supplementary Notes and Figures

Figure S1. Liquid cell setups in SEM and TEM

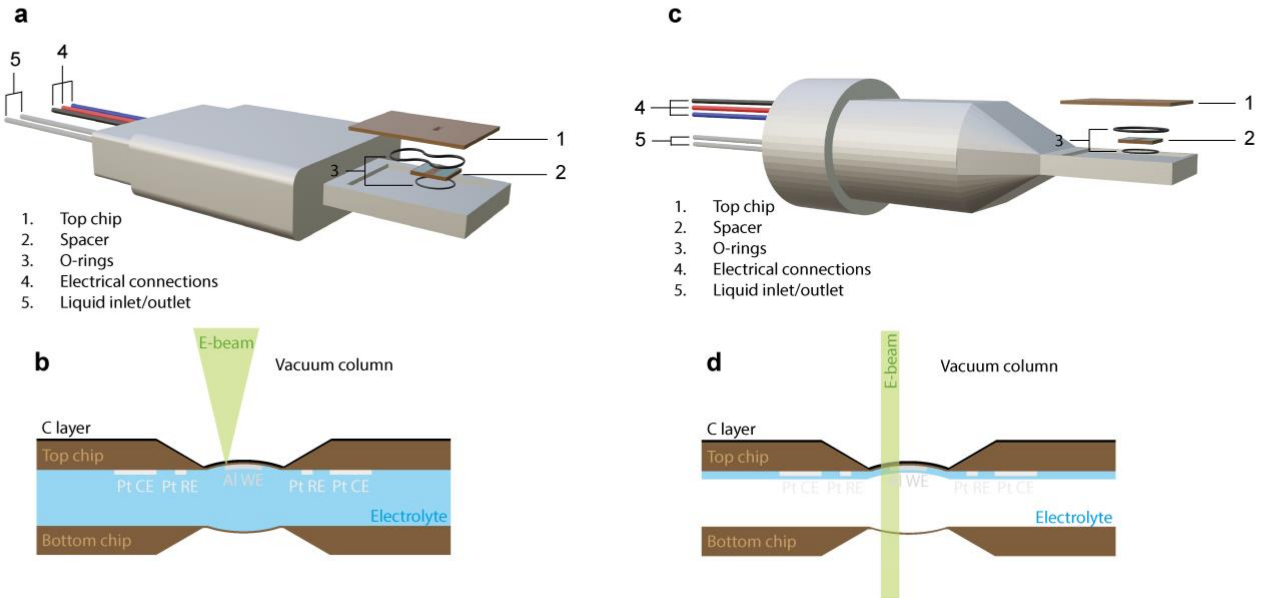

Figure S1: a) Schematic view of the SEM stage of the electrochemical liquid phase experiments and b) cross-sectional scheme of the imaging region. c) Schematic view of the TEM holder of the electrochemical liquid phase experiments and d) cross-sectional scheme of the imaging region. (Not-to-scale)

Figure S2. TEM selective area diffraction analysis of microfabricated and corroded electrochemical chips

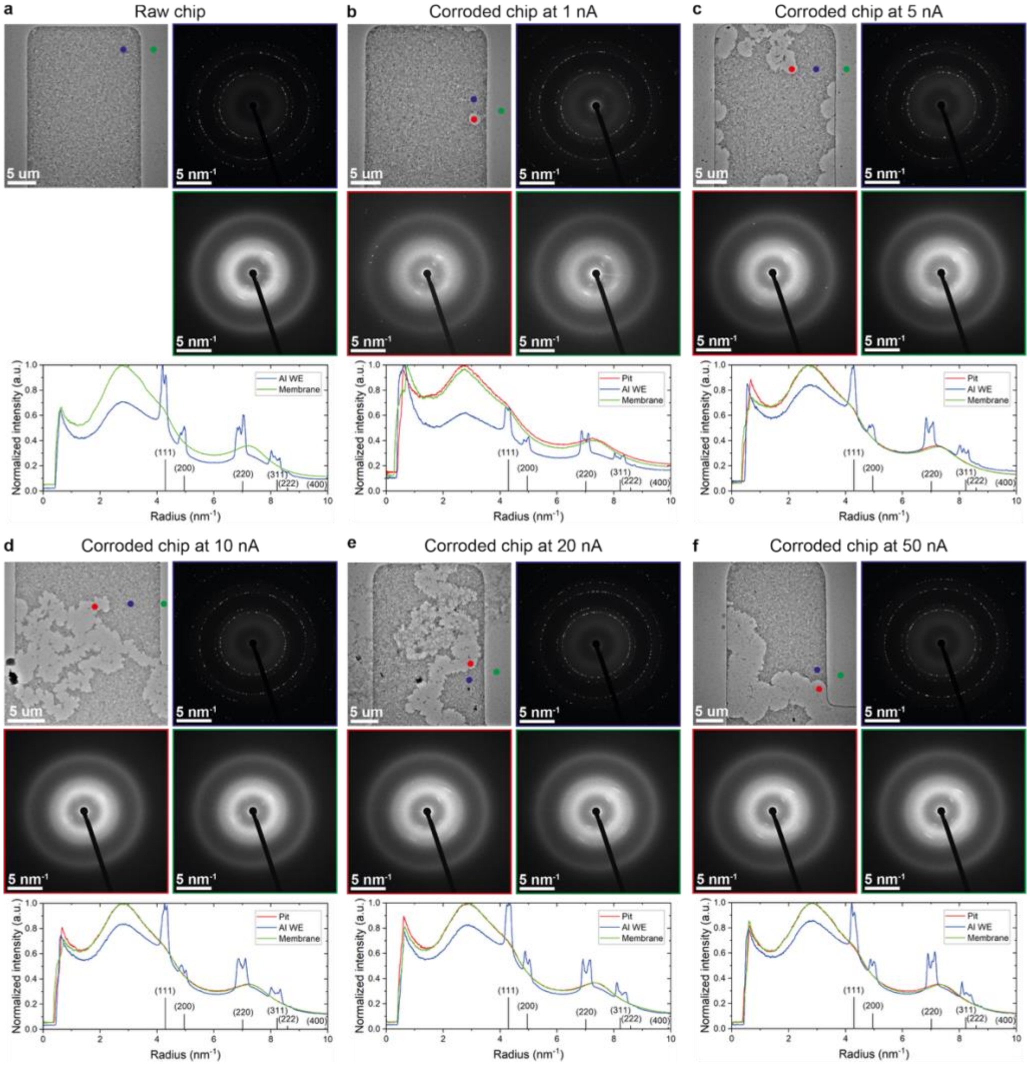

Figure S2: TEM SAED characterization of (a) a raw microfabricated chip and (b-f) the LPSEM-corroded chips at (b) 1 nA, (c) 5 nA, (d) 10 nA, (e) 20 nA, and (f) 50 nA. For each subfigure, the top left inset shows a TEM image of the studied area, the top right displays the diffraction pattern acquired on the Al WE, the center left features the diffraction pattern obtained in the pit region (when present), the center right presents the diffraction pattern acquired on the SiN_x membrane, and the bottom plot represents the radial profile of the three diffraction patterns. The locations where SAED was performed are indicated in blue for the Al WE, in green for the SiN_x membrane, and in red for the pit region, on the TEM image. For the radial profile, reference diffraction planes of Al are indicated with black lines based on the work of Mulder *et al*[1]. The measurements were performed using a 10 eV slit as energy filter. It is emphasized that a polycrystalline Al microstructure is present on the microfabricated WE, which persists after the ec-LPSEM measurements. The SiN_x membrane exhibits an amorphous diffraction pattern, as expected. Additionally, aluminum has been fully etched from the corroded region, as the diffraction patterns obtained both in the pit region and on the SiN_x membrane match.

Figure S3. Quasi-reference Pt electrode calibration

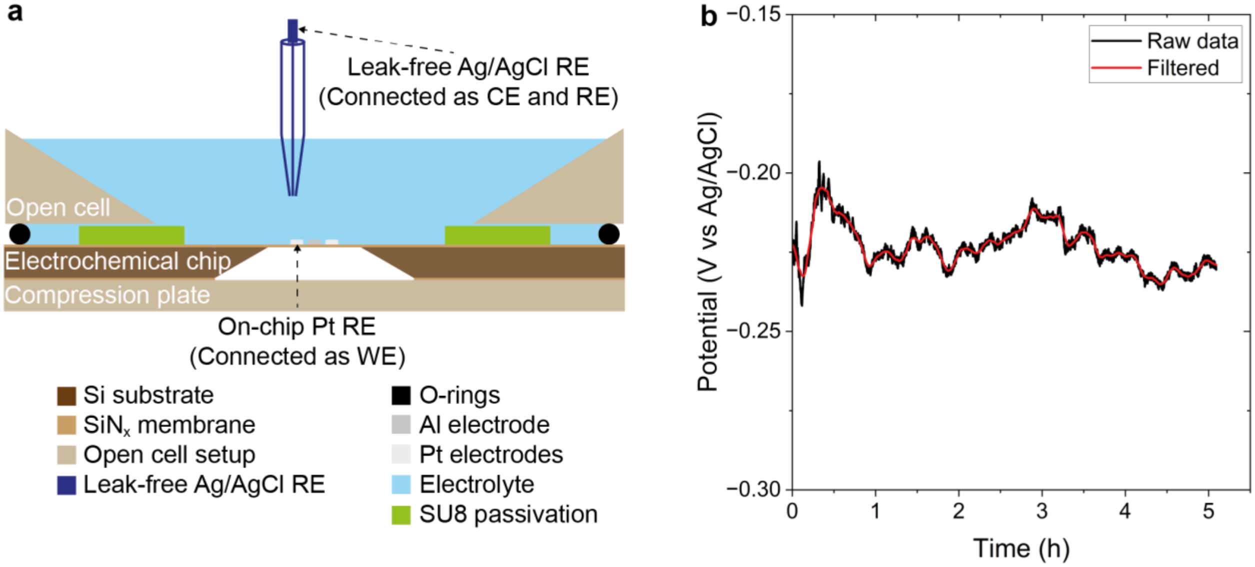

Figure S3: Electrochemical calibration of on-chip Pt quasi-reference electrode for aqueous 0.1 M NaCl solution. (a) Cross-sectional scheme of the open cell experimental setup. (b) Open-circuit voltage (OCV) measurement of the on-chip Pt quasi-reference electrode (connected as WE) vs the leak-free Ag/AgCl reference electrode (connected as CE and RE) in 0.1 M NaCl solution. The results indicate a stable potential around -0.22 V vs Ag/AgCl for the on-chip Pt quasi-reference electrode.

Note S1. LPSEM control experiments of beam-induced degradation

The effect of the electron beam on in situ measurements is a crucial consideration during liquid-phase electron microscopy experiments. To check for the role of the electron beam in our LPSEM measurements, we exposed our sample to the electron beam under similar imaging conditions as those used for the LPSEM experiments (accelerating voltage of 5kV, current probe of 64 pA, frame time of 1.64 s, 1000x magnification). The liquid cell was mounted in a similar manner to that of the LPSEM measurement and immersed in the same 0.1M NaCl electrolyte. After 15 minutes of exposure to the electron beam, no beam-induced degradation was detected, allowing us to conclude that electron beam induced degradation during LPSEM imaging was negligible.

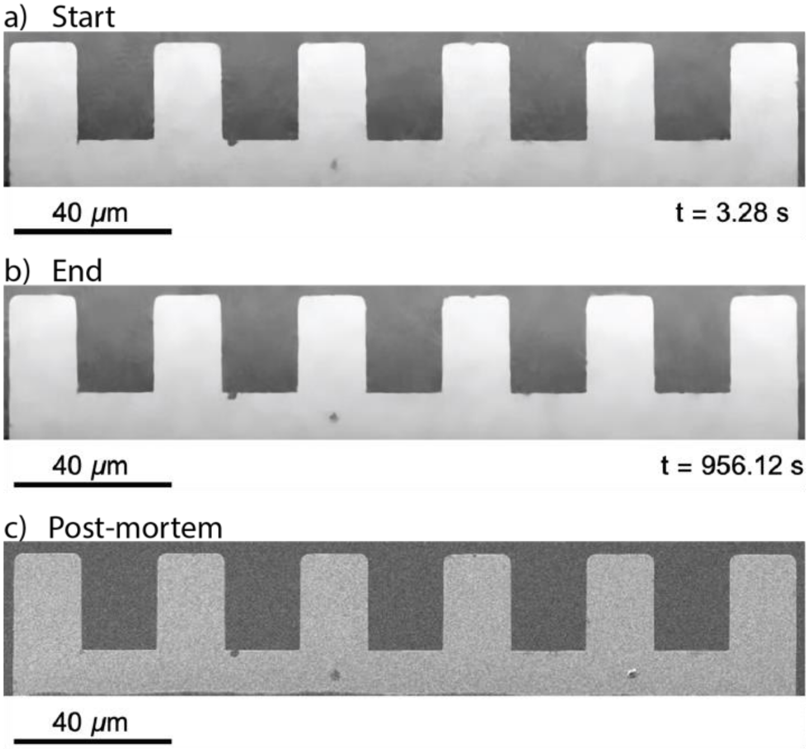

Figure S4. LPSEM images of the Al WE at the a) start and at the b) end of the control experiment. c) Post-mortem SEM image of the control experiment chip. E-beam exposure was for 15 min.

Figure S5. Denoising and segmentation pipeline

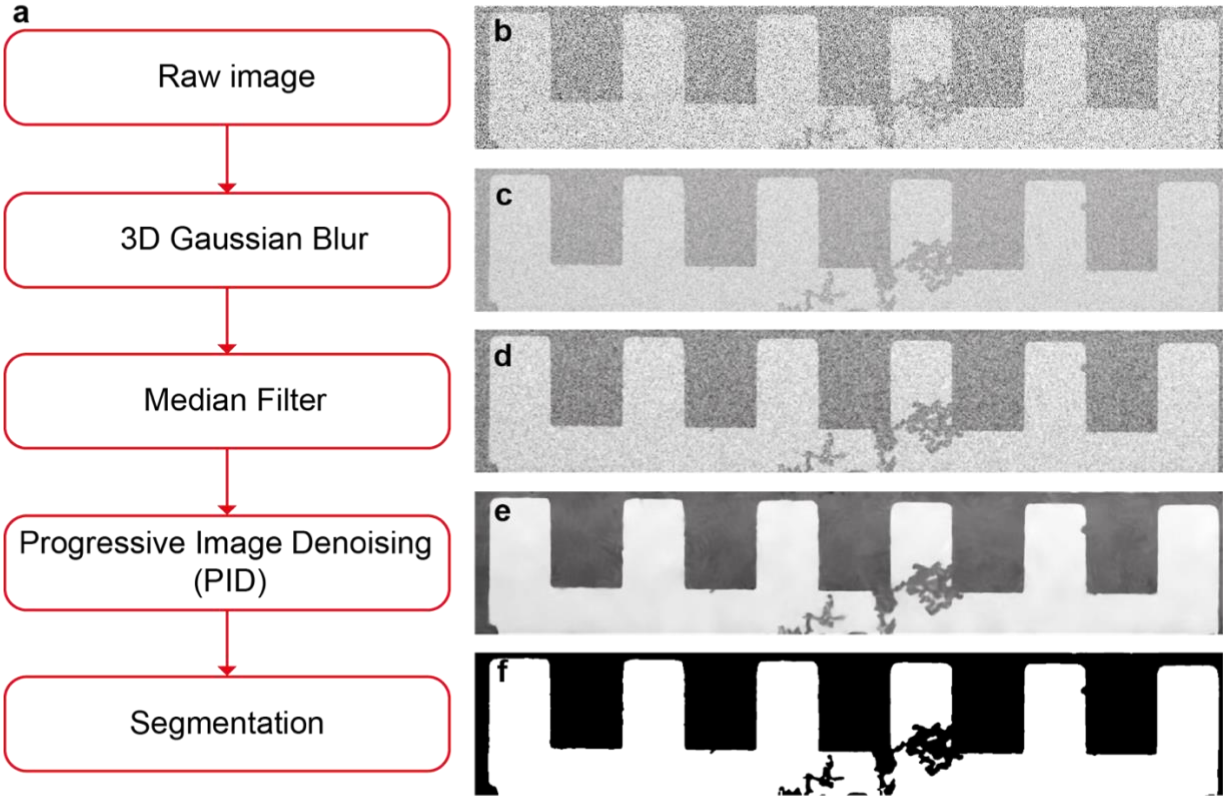

Figure S5. a) Scheme of the SEM image denoising pipeline. b) Raw SEM frame of CP measurement performed at 10 nA. c) 3D Gaussian blurred image. d) Median filtered image. e) Denoised image using *Progressive image denoising* (PID) algorithm. f) Segmented image.

Figure S6. Potentiodynamic in situ Al corrosion in the SEM

The LSV measurement was performed from - 0.1 V vs OCV to +1.5 V vs OCV at a scan rate of 1 mV/s and the experiment was stopped once the produced gas filled the imaging region. As seen in the Figure S6 below, the electrochemical signal shows a relatively constant current from -0.1 V vs OCV to -0.22 V vs Ag/AgCl, which corresponds to the potential at which a huge increase in the measured current is recorded. This potential is the pitting potential. The recorded image sequence depicts a corrosion event linked with the pitting potential. As soon as this potential was reached, the Al electrode was quickly oxidized. In fact, half of the electrode was corroded within 3 recorded frames, which corresponds to less than 5 seconds and the imaging region was filled with gas within less than 13 seconds. It is concluded that potentiodynamic measurements in LPSEM proceed too rapidly and details on the early stages of localized corrosion are not possible under these electrochemical conditions.

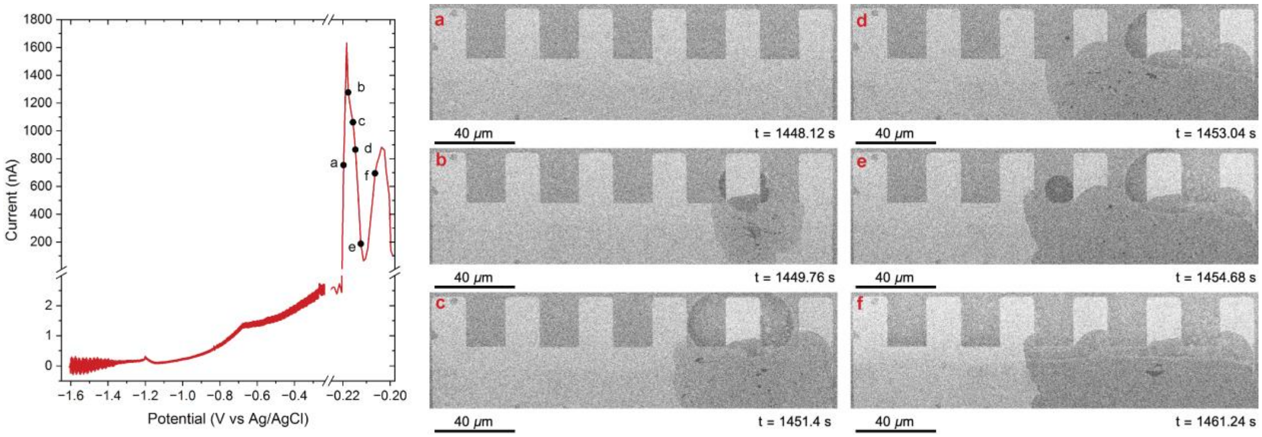

Figure S6. Time series of LSV measurement performed from -0.1 V vs OCV to 1.5 V vs OCV in the SEM. The left plot corresponds to the electrochemical signal recorded during the acquisition and the right images correspond to in situ acquired SEM images. The black dots on the electrochemical signal match the time of each SEM image.

Figure S7. Corrosion events on the full imaged window for CP at 1 nA

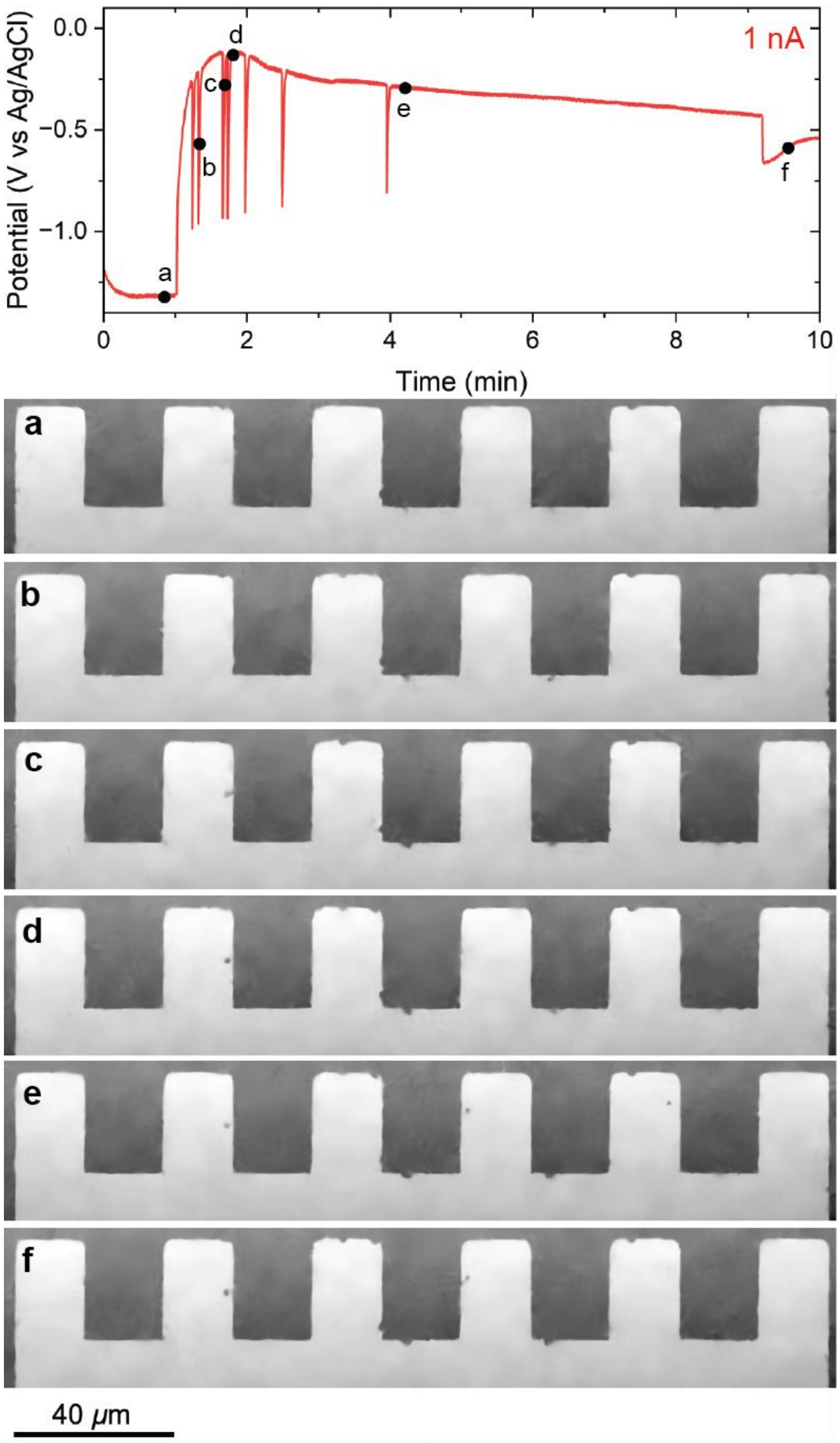

Figure S7: Time series of CP measurement performed at 1 nA in the SEM. The top plot corresponds to the electrochemical signal recorded during the acquisition and the bottom images correspond to in situ acquired SEM images. The black dots on the electrochemical signal matches the time of each SEM image.

Figure S8. Corrosion events on the full imaged window for CP at 5 nA

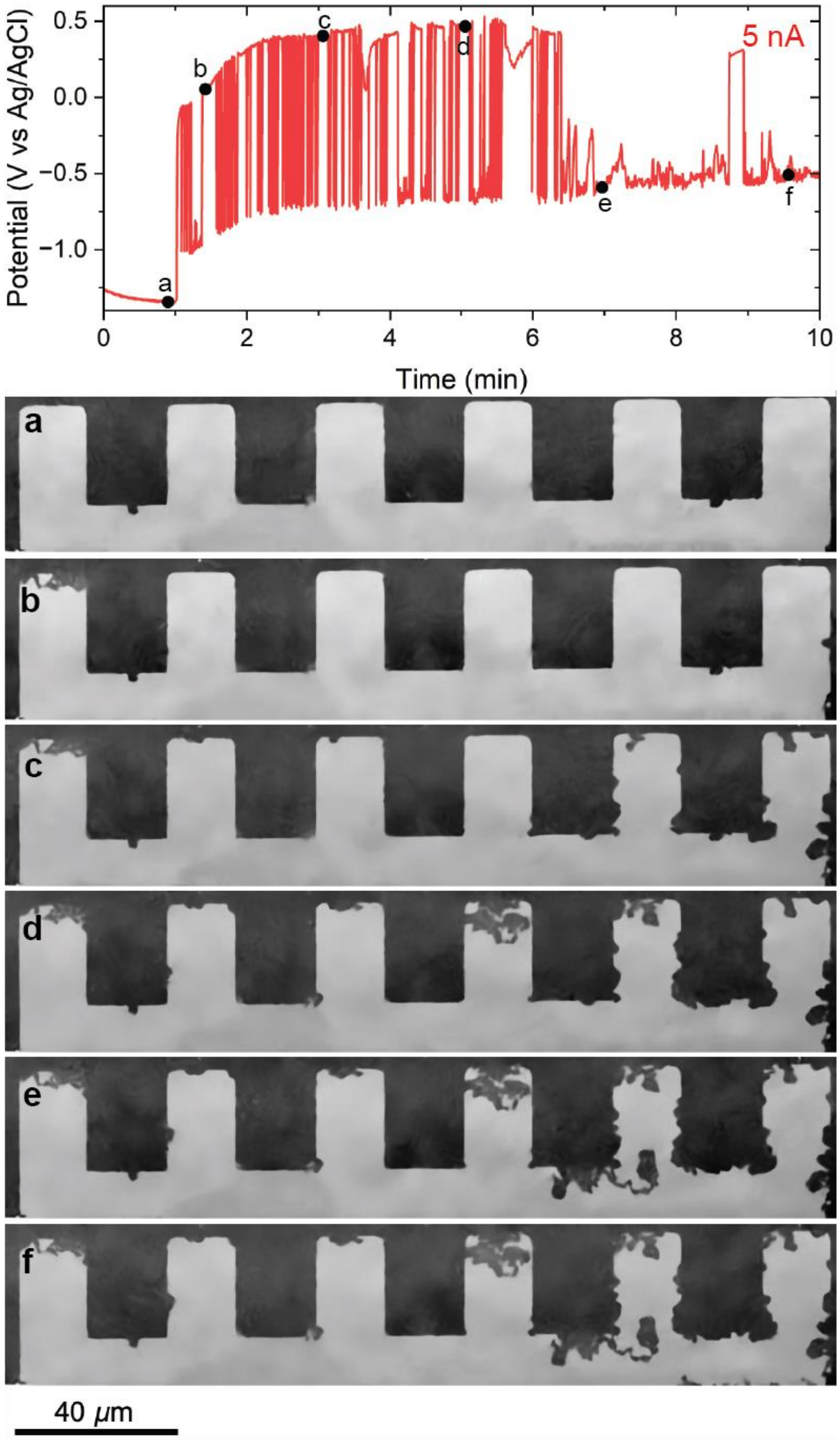

Figure S8: Time series of CP measurement performed at 5 nA in the SEM. The top plot corresponds to the electrochemical signal recorded during the acquisition and the bottom images correspond to in situ acquired SEM images. The black dots on the electrochemical signal matches the time of each SEM image.

Figure S9. Corrosion events on the full imaged window for CP at 10 nA

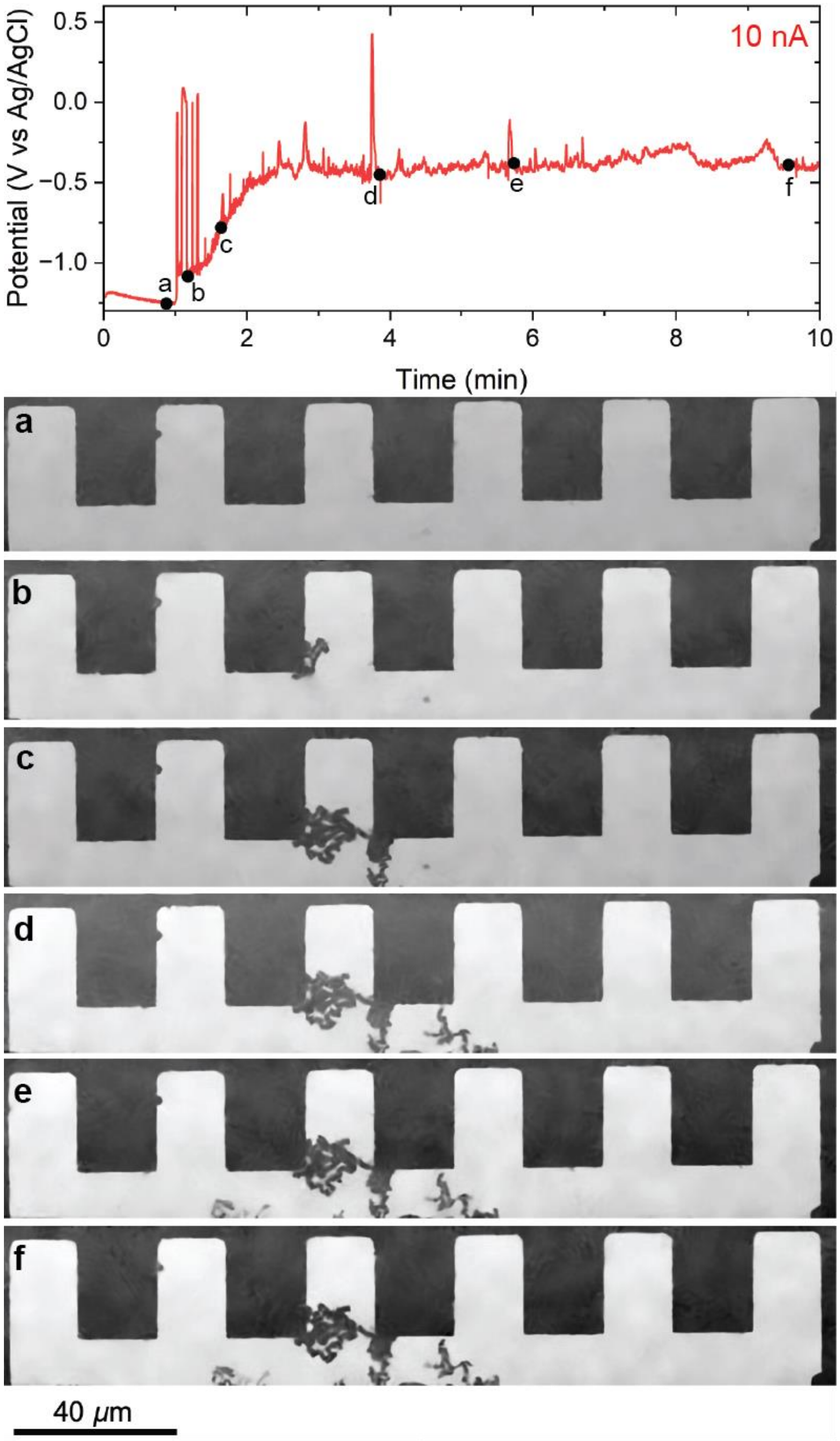

Figure S9: Time series of CP measurement performed at 10 nA in the SEM. The top plot corresponds to the electrochemical signal recorded during the acquisition and the bottom images correspond to in situ acquired SEM images. The black dots on the electrochemical signal matches the time of each SEM image.

Figure S10. Corrosion events on the full imaged window for CP at 20 nA

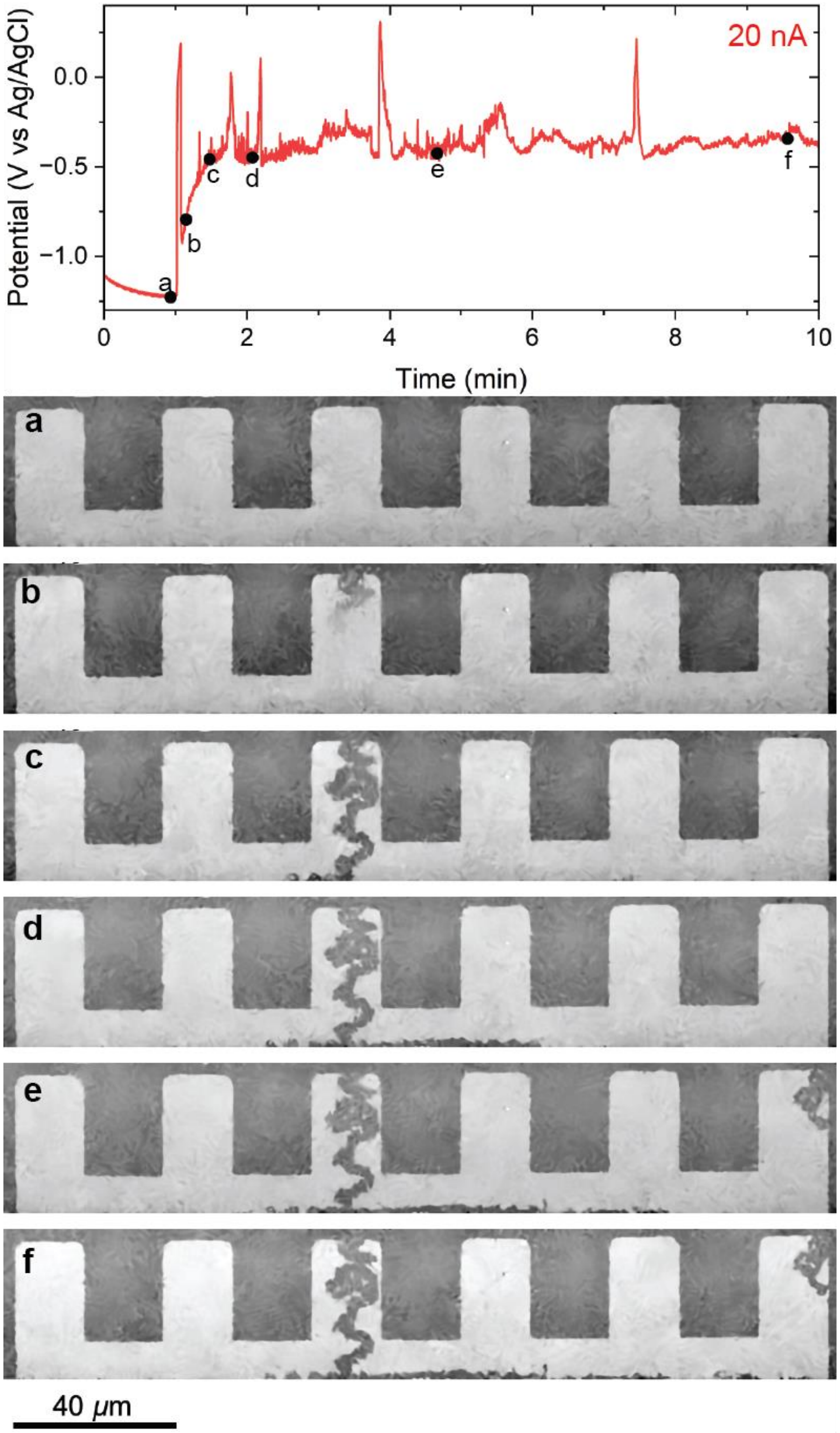

Figure S10: Time series of CP measurement performed at 20 nA in the SEM. The top plot corresponds to the electrochemical signal recorded during the acquisition and the bottom images correspond to in situ acquired SEM images. The black dots on the electrochemical signal matches the time of each SEM image.

Figure S11. Corrosion events on the full imaged window for CP at 50 nA

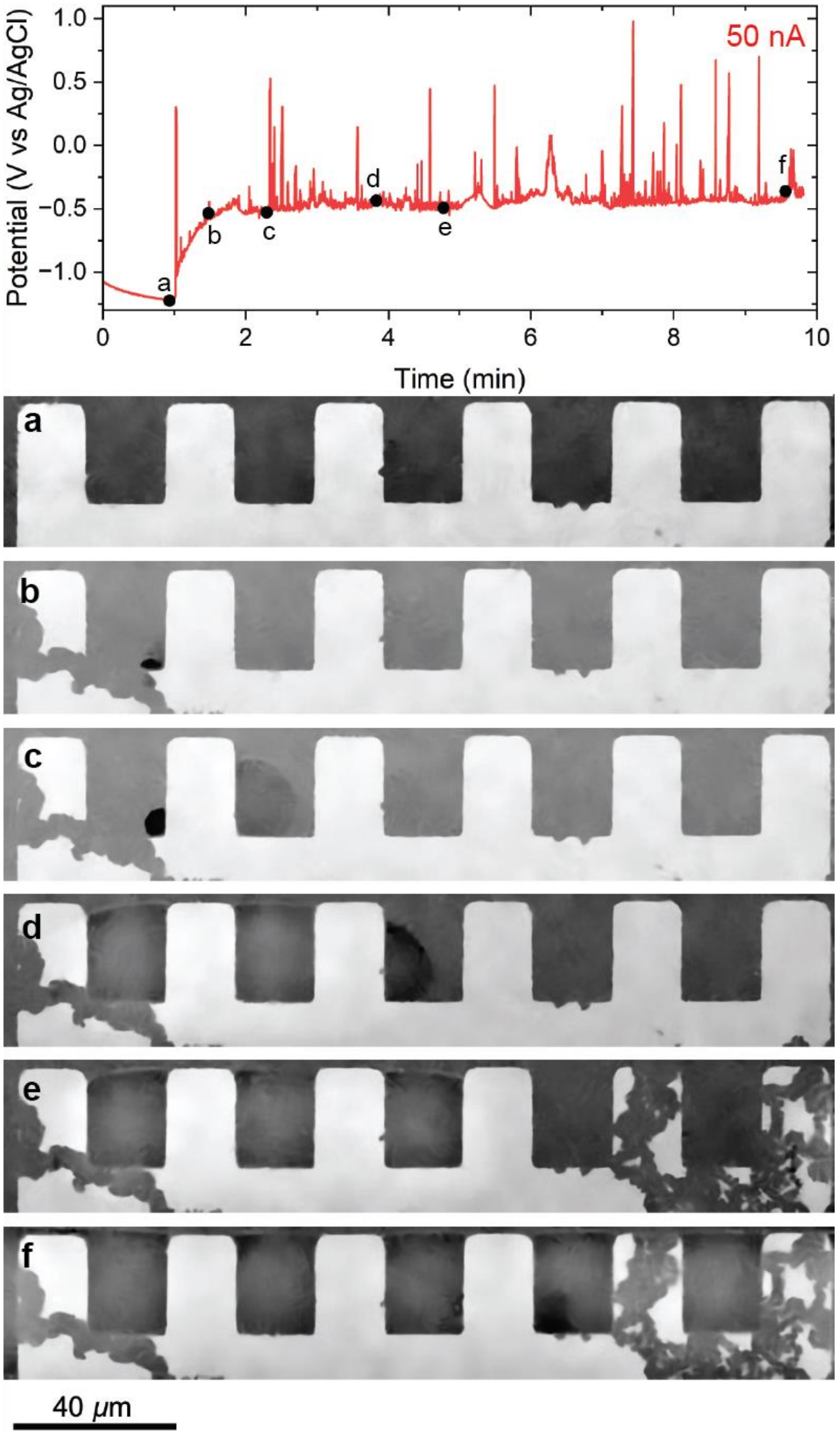

Figure S11: Time series of CP measurement performed at 50 nA in the SEM. The top plot corresponds to the electrochemical signal recorded during the acquisition and the bottom images correspond to in situ acquired SEM images. The black dots on the electrochemical signal matches the time of each SEM image.

Note S2. Reliability of the in situ galvanostatic measurements

To ensure the meaningfulness of the in situ galvanostatic measurements, their reproducibility was assessed by performing similar electrochemical measurements within different experimental setups and by comparing the obtained response signal. Chronopotentiometry experiments were performed at 20 nA in an open cell experimental setup as described in Figure S12a, and in the SEM holder (Figure S12b,c), both on the bench and in situ. All experiments were performed in 0.1 M NaCl aqueous electrolyte. The CP measurement in the open cell setup was performed using the on-chip Al electrode as the WE, a Pt wire as CE, and a leak-free Ag/AgCl reference electrode. The CP measurements conducted in the LPSEM holder were performed using the on-chip Al electrode as the WE, the on-chip external Pt electrode as CE, and the second on-chip Pt electrode as RE. The measurements performed in the LPSEM holder were transposed relative to a Ag/AgCl reference potential following the calibration presented in Figure S3. The results of the CP measurements in the different setups are presented in Figure S12d.

The three curves depict a similar global behavior. Initially, there is a peak in the potential as soon as the anodic current is applied, corresponding to galvanostatic charging[2–4]. After the consecutive sharp potential decay, a potential plateau corresponding to the Al oxidation is observed. A discrepancy of about 250 mV was observed for this potential plateau between the measurement performed in the open cell setup (black curve in Figure S12d) and the measurements performed in the LPSEM holder (red and blue curves in Figure S12d). This discrepancy could be explained by the change in the electrode geometry, which is coplanar in the SEM holder, the confined volume of the liquid cell, or the change in the distances between the different electrodes, and thus the change in ohmic drop, of the 3-electrode system between the two setups. Direct contact of the electrolyte with the ambient air within the open-cell experimental setup could also alter the results compared to those obtained in the SEM holder, where the solution is relatively de-aerated.

Another discrepancy between the two types of measurements comes from the region between the galvanostatic charging peak and the oxidation plateau. A direct transition is observed in the experiment performed in the open cell setup, whereas a transitory regime is depicted for the measurement performed in the LPSEM holder. This is likely due to the difference in the type of reference electrode used in the different setups. The Pt quasi-reference used as RE for the LPSEM holder measurements may require stabilization, which could account for the transient regime observed in the red and blue curves in Figure S12d.

Finally, the measurements performed in the LPSEM holder show a similar potential trend for the Al oxidation plateau. This highlights the limited effect of the electron beam on the SEM measurements conducted in this study.

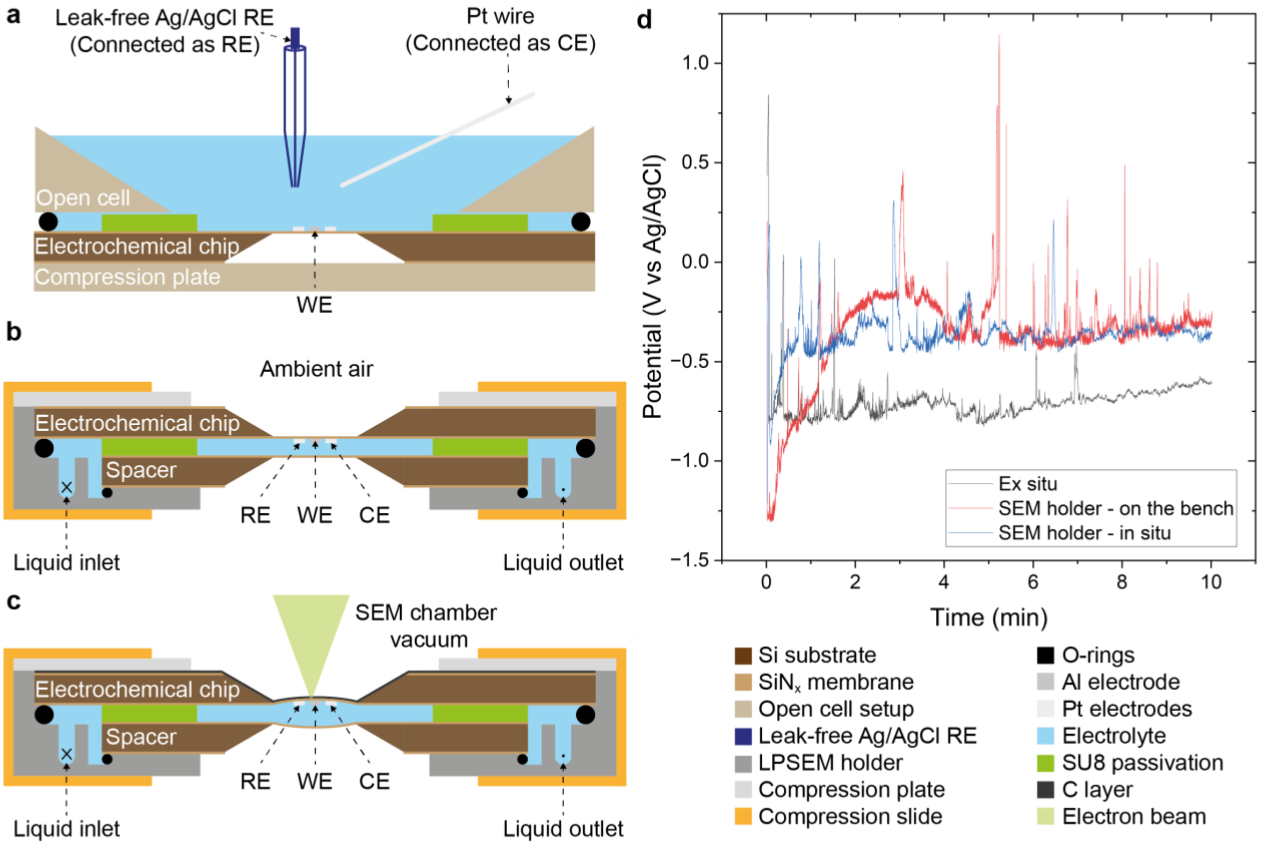

Figure S12: Comparison of chronopotentiometry measurements upon different experimental setups. (a,b,c) Cross-section schemes of the (a) open cell, (b) on-bench LPSEM holder, and (c) in situ LPSEM holder experimental setups. (d) CP measurements performed at 20 nA in 0.1 M NaCl electrolyte in the open cell experimental setup (black) and in the LPSEM holder, on the bench (red), and in situ (blue).

Note S3. Electron dose calculation

The impact of the electron beam on the in situ measurements is an important concern when performing liquid-phase electron microscopy experiments. Indeed, it might trigger side reactions such as radiolysis of the aqueous environment, that might directly hinder the chemical processes under investigation within the liquid cell[5,6]. Herein, we address that problem by using a limited dose during the experiment, and by performing control experiments in the microscope, as shown in Notes S1 and S2. Electron doses were calculated with expressions derived by Egerton[7].

SEM experiments were performed at 5 kV, with a current probe of $I_b = 64$ pA, a frame time $t_f = 1.64$ s, at a magnification of 1000x corresponding to a frame area A of 207.57 x 150.41 μm^2 for 11 min in total (i.e. number of frames $n_f = 405$). Based on Equations 1 and 2, this corresponds to a dose per frame D_f of 0.021 e^-/nm^2, a dose rate of 0.013 $e^-/(nm^2 \cdot s)$, and a total irradiation dose D_t of 8.50 e^-/nm^2. Contrary to transmission techniques, this dose is only valid for the surface of the cell exposed to the electron beam, as the electrons do not transmit through the full cell. Furthermore, the membrane and electrode being only a part of the field of view at this magnification, it is very likely that the actual dose perceived by the sample is smaller than the calculated one.

$$D_f = \frac{I_b}{A} t_f \qquad \text{Equation 1}$$

$$D_t = \frac{I_b}{A} t_f \cdot n_f \qquad \text{Equation 2}$$

TEM experiments were performed at 200 kV, with a measured current screen of $I_b = 2.08$ nA, an exposure time of $\Delta t = 500$ ms, at a magnification of 2600x using an image size of 4096 x 4096 pixels, with a pixel size of 6.082 nm for $n_f = 159$ frames. Assuming that the diagonal of the acquired image is the outside diameter d of the beam (which is overestimating the actual dose), and based on Equations 3 and 4, this corresponds to a dose per frame D_f of 6.66 e^-/nm^2, a dose rate of 13.3 $e^-/(nm^2 \cdot s)$ and a total irradiation dose D_t of 1058.8 e^-/nm^2.

$$D_f = \frac{4}{\pi} \frac{I_b}{d^2} \Delta t \qquad \text{Equation 3}$$

$$D_t = \frac{4}{\pi} \frac{I_b}{d^2} \Delta t \cdot n_f \qquad \text{Equation 4}$$

The TEM dose rate used within these experiments is thus 1000 times higher than the SEM one, likely explaining the discrepancies induced by the electron beam at the TEM imaging scale.

Note S4. Monte-Carlo simulation of e-beam irradiated liquid cells

Monte Carlo simulations were performed using Casino v2.48 software to determine the origin of the electrons detected in the sample during in situ SEM imaging. For this purpose, the liquid cell was modelled as a stack comprising a 5 nm carbon (C) layer, a 50 nm silicon nitride (SiN_x) membrane, an aluminium working electrode (Al WE) with thicknesses of 50, 30, 10 and 0 nm, and a 1000 nm layer of water (H_2O). The microscope conditions used for the simulations consisted of an acceleration voltage of 5 keV and a spot size of 2 nm, with 100,000 electrons involved in the simulations.

Figures S13a–d show the trajectories of the simulated electrons within the liquid cells as the thickness of the aluminium working electrode (Al WE) decreases. Figures S13e-h show the maximum depth of electrons, highlighted by the red trajectories in Figures S13a-d. The results suggest that the majority of electrons that escape the liquid cell, and are thus detected in SEM imaging, originate from the Al WE, followed by the SiN_x membrane once the Al WE is absent. Electrons emitted back from the liquid water contribute only a small portion of the signal. Even as the Al WE thickness decreases, the electron signal emitted back in the chamber primarily arises from the metallic layer. This explains why pits growing from the Al-electrolyte interface are detected only after the Al thickness has been completely corroded; before that, electrons from the remaining Al layer dominate the back-emitted signal.

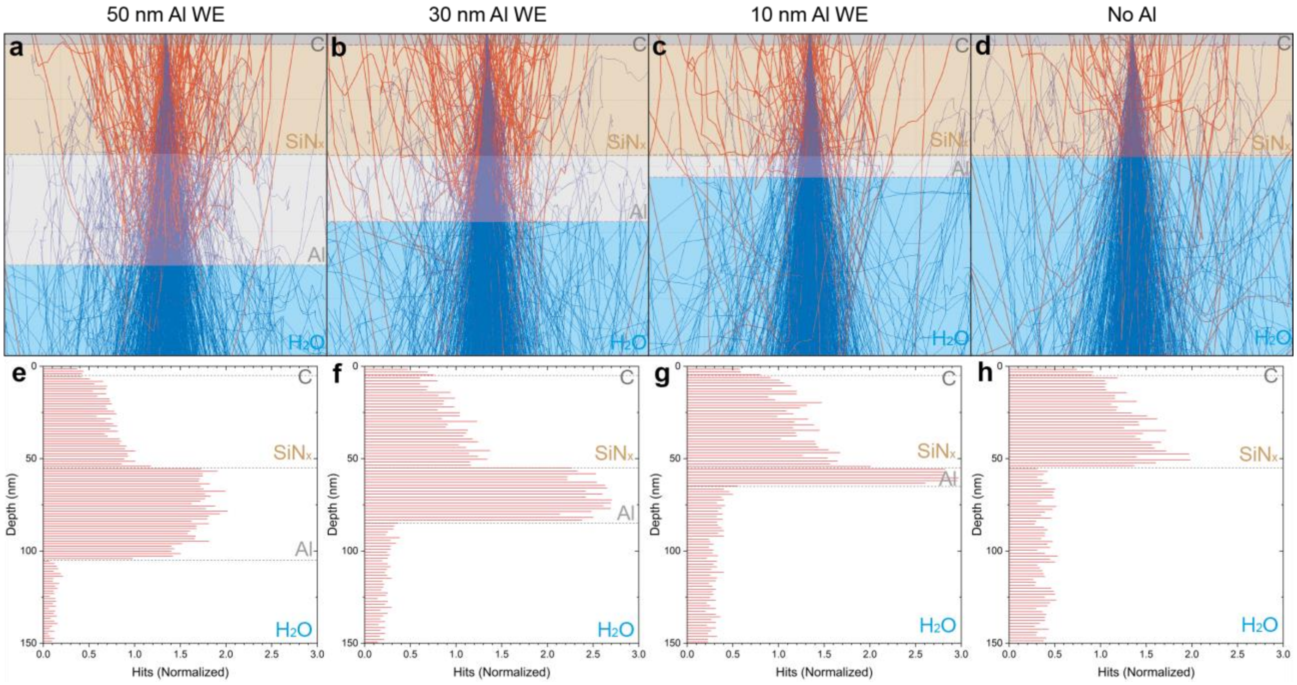

Figure S13: Monte-Carlo simulation (Casino v2.48) of e-beam irradiated liquid cells with decreasing Al WE thickness. (a-d) Trajectories of the simulated electrons within the liquid cell. Blue trajectories ends up in the sample, whereas red trajectories escape the specimen and can thus be detected. (e-h) Maximum depth of the electrons escaping the liquid cells. Simulations were performed for Al WE with a thickness of (a,e) 50 nm, (b,f) 30 nm, (c,g) 10 nm, and (d,h) no Al WE remaining on the SiN_x membrane.

Figure S14. Post-mortem optical images of LPSEM corroded electrochemical chips

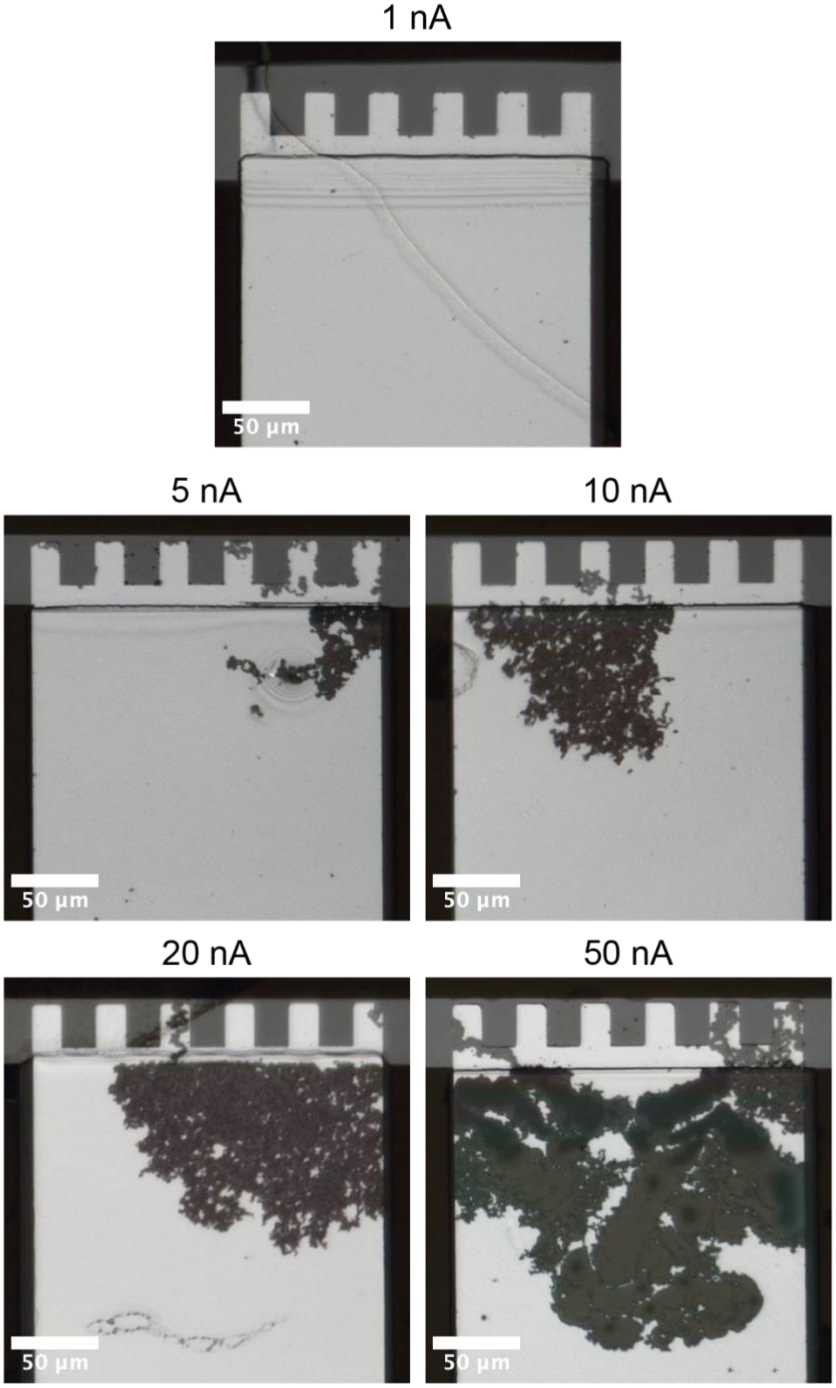

Figure S14: Post-mortem optical images of the corroded electrochemical chips during the LPSEM experiments. The anodic current applied for the chronopotentiometry measurements is indicated above each image.

Note S5. In situ TEM CP and consecutive EELS measurement for molecular hydrogen detection

We performed the 50 nA CP measurement in the TEM, this time without imaging while the electrochemistry was taking place, to avoid any beam-induced artifact. The goal was to reproduce the final situation where a gas bubble was filling the whole imaging region in the SEM. The electrochemical profile is shown in Figure S15a. Despite a huge increase in the potential at the start of the CP measurement, no galvanostatic charging peak was observed in the electrochemical curve. The kinetic of this galvanostatic charging likely was too fast to be properly recorded by the electrochemical setup. However, the potential plateau of the active anodic oxidation matches the previous measurements well. When imaging the sample in ADF mode, most of the field of view depicted a darker contrast, surrounded in some areas with a bright region corresponding to the liquid surrounding the electrochemically formed gas bubble (Figure S15d). STEM EELS was then performed in the gas bubble, both on the Al electrode and the SiN_x membrane and compared to similar measurements done previously in vacuum. EELS measurements on the Al electrode show a very sharp plasmonic peak, with a maximum at around 15 eV, associated with the Al volume plasmon[8]. The drawback here is that the ionisation edge of hydrogen is located at the onset of this plasmonic peak. It is consequently difficult to verify any hydrogen formation on the electrode itself. However, the SiN_x membrane does not show a similar plasmonic peak, making it more suitable for observing the presence of hydrogen in the system. Thus, EELS measurement on the membrane revealed a small feature at 12.5 eV, corresponding to the hydrogen ionisation edge.

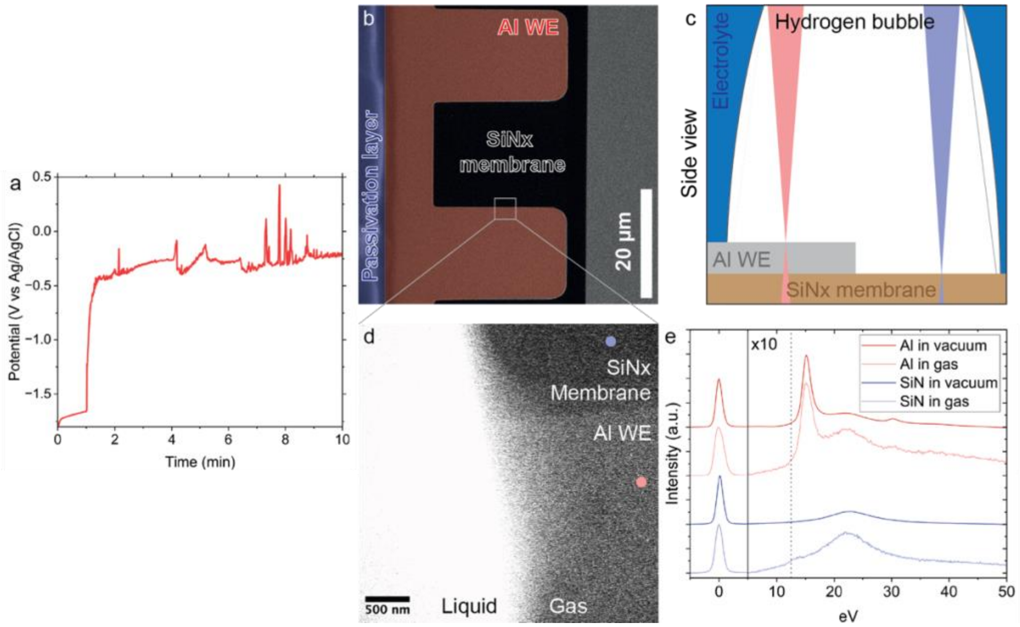

Figure S15: CP performed at 50 nA in the TEM without imaging and consecutive EELS measurement. (a) Electrochemical curve. (b) SEM image of the location of the region used for EELS acquisition. (c) Side view schematic of the region used for EELS acquisition. (d) ADF image of the region used for EELS acquisition. (e) EELS measurement. The colored spot highlighted in (c,d) corresponds to the Al and SiN spectra acquired in gas in (e). For (e), EELS spectra were normalized such that the maximum value of the zero loss peak is equal for each spectrum. Also in (e) the straight line at 5 eV corresponds to the demarcation line after which spectra intensity was multiplied by 10 and the dotted line corresponds to an energy of 12.5 eV.

Figure S16. AFM post-mortem characterization of the LPSEM-corroded chips

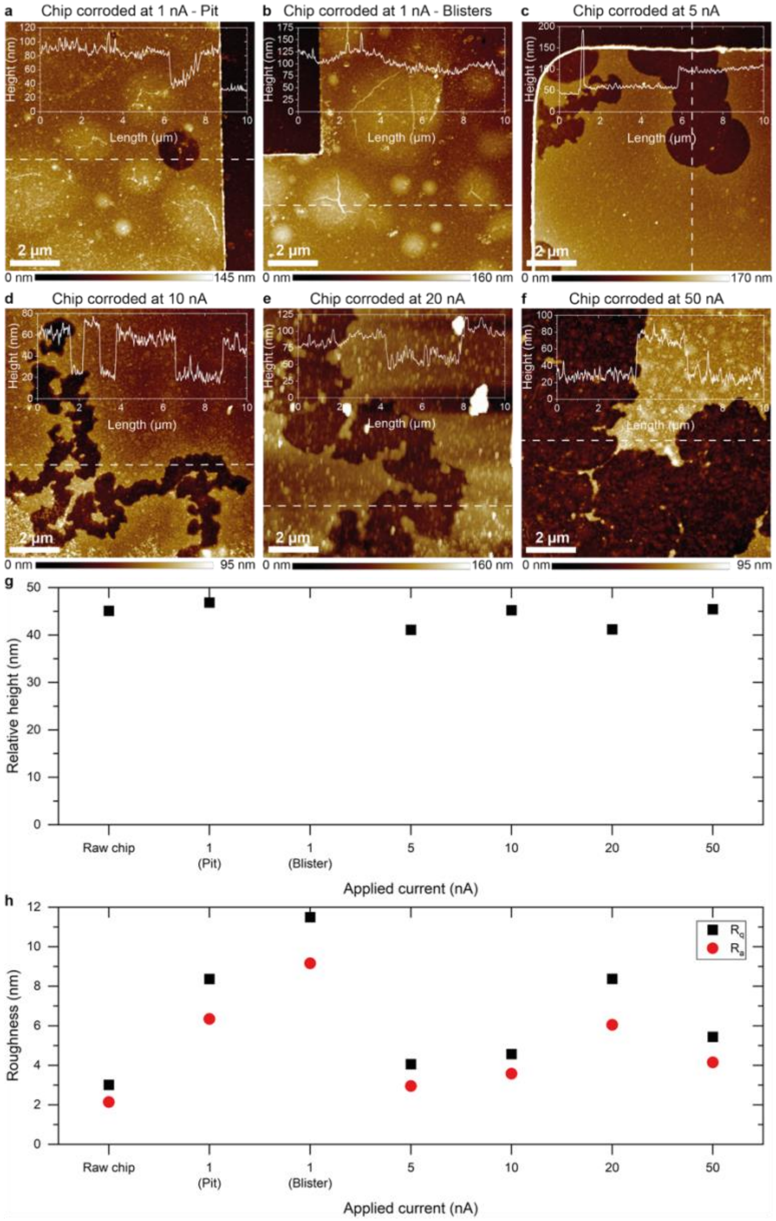

Figure S16: AFM post-mortem characterization of the LPSEM-corroded chips. (a-f) Height map of the chips corroded during the LPSEM CP measurement performed at (a,b) 1 nA, (c) 5 nA, (d) 10 nA, (e) 20 nA, and (f) 50 nA. For each map, a line profile taken from the dashed line is displayed on the inset plot. Subfigure (a) shows a region where a pit formed on the Al electrode, while (b) presents an area with a high concentration of blisters for the chip corroded at 1 nA. (g) Plot of the relative height between the corroded region and the remaining aluminum layer. The measured relative height is in the same range than the initial aluminum thickness, highlighting the removal of the whole aluminum thickness in the pitted regions. (h) Plot of the roughness of the Al electrode as a function of the applied anodic current of the LPSEM CP measurement. All corroded chips depict a higher roughness than the microfabricated one. The larger discrepancies measured for the chip corroded at 1 nA can be attributed to the presence of blisters, as illustrated in AFM maps (a) and (b). In contrast, the inadequate cleaning of the chip corroded at 20 nA likely accounts for its increased roughness.

References

1. Mulder, F. M. *et al.* Hydrogen in the Metal−Organic Framework Cr MIL-53. *J. Phys. Chem. C* **114**, 10648–10655 (2010).
2. Stevanović, R. M., Despić, A. R. & Dražić, D. M. Activation of aluminium in chloride containing solutions. *Electrochimica Acta* **33**, 397–404 (1988).
3. Despić, A. & Parkhutik, V. P. Electrochemistry of Aluminum in Aqueous Solutions and Physics of Its Anodic Oxide. in *Modern Aspects of Electrochemistry No. 20* (eds. Bockris, J. O., White, R. E. & Conway, B. E.) vol. 20 401–503 (Springer US, Boston, MA, 1989).
4. Smoljko, I., Gudić, S., Kuzmanić, N. & Kliškić, M. Electrochemical properties of aluminium anodes for Al/air batteries with aqueous sodium chloride electrolyte. *Journal of Applied Electrochemistry* **42**, 969–977 (2012).
5. Han, C., Islam, M. T. & Ni, C. In Situ TEM of Electrochemical Incidents: Effects of Biasing and Electron Beam on Electrochemistry. *ACS Omega* **6**, 6537–6546 (2021).
6. Robberstad Møller-Nilsen, R. E. *et al.* Quantifying Aqueous Radiolytic Products in Liquid Phase Electron Microscopy. *Journal of Physical Chemistry C* **127**, 15512–15522 (2023).
7. Egerton, R. F. Dose measurement in the TEM and STEM. *Ultramicroscopy* **229**, 113363 (2021).
8. Stöckli, T., Bonard, J.-M., Stadelmann, P.-A. & Châtelain, A. EELS investigation of plasmon excitations in aluminum nanospheres and carbon nanotubes. *Zeitschrift für Physik D Atoms,Molecules and Clusters* **40**, 425–428 (1997).
